# Lipidomic Screening of Marine Diatoms Reveals Release of Dissolved Oxylipins Associated with Silicon Limitation and Growth Phase

**DOI:** 10.3390/md23110424

**Published:** 2025-10-31

**Authors:** Imanol Ulloa, Jiwoon Hwang, Matthew D. Johnson, Bethanie R. Edwards

**Affiliations:** 1 Department of Earth and Planetary Science, University of California Berkeley, Berkeley, CA 94720, USA; jiwoon.hwang@berkeley.edu; 2 Biology Department, Woods Hole Oceanographic Institution, Falmouth, MA 02543, USA; mattjohnson@whoi.edu

**Keywords:** diatom, oxylipin, linear oxygenated fatty acid, lipidomics

## Abstract

Marine diatoms are an important group of phytoplankton that can shape marine ecosystems and global carbon cycling. When stressed, either physiologically or by grazing, diatoms release oxidized, lipid-derived signals known as oxylipins. Diatom-derived oxylipins are proposed to serve as defense and signaling chemicals that affect multiple components of marine ecosystems. Therefore, to elucidate the diversity of diatom-derived oxylipins produced during stress, we profiled the spectrum of dissolved lipids of five diatom species in culture under silicon limitation and across growth phases using ultra-high performance liquid chromatography coupled with high-resolution accurate mass spectrometry. In this study, we present evidence that physiological changes associated with Si-limitation elicit the extracellular release of linear oxygenated fatty acids (LOFAs) across five diatom species. For diatoms like *Skeletonema japonicum* and *Pseudo-nitzschia multiseries*, silicon limitation induced a distinct lipidomic signature driven by oxylipins known to be allelopathic. While their lipoxygenases were found to be different, *S. japonicum* and *P. multiseries* had the most similar dissolved lipidomes, suggesting alternative controls on oxylipin biosynthesis. Consequently, elevated oxylipin concentrations with silicon stress, estimated up to 5.91 µM, pose implications for diatoms at sea, potentially affecting ecosystems and biogeochemistry.

## 1. Introduction

Marine phytoplankton account for a substantial portion of primary production [1,2] that is transferred up marine food webs, exported to ocean depths as particulate organic matter (POM) [3,4], or released as dissolved organic matter (DOM). Release of DOM from live phytoplankton can serve multiple roles, acting as mechanisms for carbon excretion in the form of transparent exopolymer particles (TEP) [5], but also as a dispersal mechanism for signaling and defense chemicals that can shape marine ecosystems and biogeochemistry overall [6,7]. In particular, diatoms release lipid-derived compounds known as oxylipins that are bioactive in other phytoplankton [8], zooplankton [9,10,11], and bacteria [12,13,14]. First documented as defense chemicals that affect copepod reproduction [9,11], *Diatom*-derived oxylipins have since been proposed to act as signaling chemicals between diatoms [15,16] and allelochemicals impacting microbes within the phycosphere [14].

Oxylipin biosynthesis is mediated by multiple enzymes, resulting in two major groups: polyunsaturated aldehydes (PUA) and linear oxygenated fatty acids (LOFAs). PUA production in diatoms generally occurs under stress or when cell membranes are disrupted [15,17,18,19]. PUAs are associated with reduced copepod hatching success [9,11] and are proposed to regulate diatom cell death during unfavorable conditions [15]. PUAs can also play a role in marine biogeochemistry, affecting the remineralization of POM by stimulating bacterial respiration [13,20]. LOFAs, the precursors to PUAs, are also released during cell membrane disruption [21], as well as during viral infection [16], in the presence of an algicidal bacterium [14], and at the end of diatom blooms [22]. In field observations, LOFAs were confirmed to be mainly produced by diatoms, and concentrations per cell were inversely correlated with diatom density, suggesting a chemical signaling role [23]. LOFAs are also thought to serve as a form of chemical defense by affecting copepod reproduction [22,24,25], microzooplankton grazing [10,26], and growth of the algicidal bacterium, *Kordia algicida* [14]. Considering that LOFAs are important secondary metabolites in diatoms, more information is needed regarding their chemical diversity across different stressors to better understand their role in marine ecosystems.

Thus, to elucidate the lipid plasticity of diatom-derived oxylipins produced during stress, we profiled the spectrum of dissolved lipids (the dissolved lipidome) of five diatom cultures under silicon limitation (Si-limitation) and across growth phase (Table 1). We investigated these effects on two bloom-forming diatoms commonly found in the Pacific Ocean, *Skeletonema japonicum* and *Pseudo-nitzschia multiseries.* The model PUA producer, *Thalassiosira rotula*, was also investigated in addition to two diatoms isolated from the San Pedro Ocean Time Series (SPOT) off the coast of California, USA. One of the SPOT isolates was identified as a *Chaetoceros* species, which are ubiquitous across the global ocean (Table 1). Previous observations with *Skeletonema marinoi* have observed PUA release with culture decline [19] and nutrient limitation, especially silicon [18]. However, the effect of Si-limitation on the release of LOFAs remains underexplored. By leveraging lipidomics, we provide insights into the fatty acid and LOFA diversity of marine diatoms, some of which could serve as potential stress markers and candidates for assessing chemical signaling in marine ecosystems.

## 2. Results and Discussion

### 2.1. Identification of Fatty Acids and Oxylipins

A total of 5852 features were identified and a subset was annotated via LOBSTAHS [42] as free fatty acids (FFAs), polyunsaturated aldehydes (PUAs), intact monoacylglycerols (IP-MAGs), and intact diacylglycerols (IP-DAGs). In this workflow, adduct ion formation patterns were used to assign putative annotations. As a result, eicosapentaenoic acid (EPA) was annotated as a free fatty acid with 20 carbons and 5 double bonds in the following format: FFA 20:5. Oxidized fatty acids were further denoted with (+ #O), depending on the number of oxygen insertions. Of the identified features, 4.37% were putatively annotated as FFAs and PUAs (*n* = 256). Annotations as FFAs were then retained for manual verification, resulting in 60 features with high-quality extracted ion chromatograms. Of the 60 features annotated by LOBSTAHS, 6 were further confirmed as LOFAs using MS2 fragmentation provided by in silico and standard spectral databases. Five additional oxylipins were identified and confirmed only through the MSDIAL workflow [43] (Table 2). Further details on the annotation process are discussed in the methods section. MS2 fragmentation for each compound can be found in the Supplementary Materials (Figures S1–S16).

### 2.2. Increased Release of Dissolved Oxylipins and Fatty Acids with Si-Limitation

**Figure 1:**
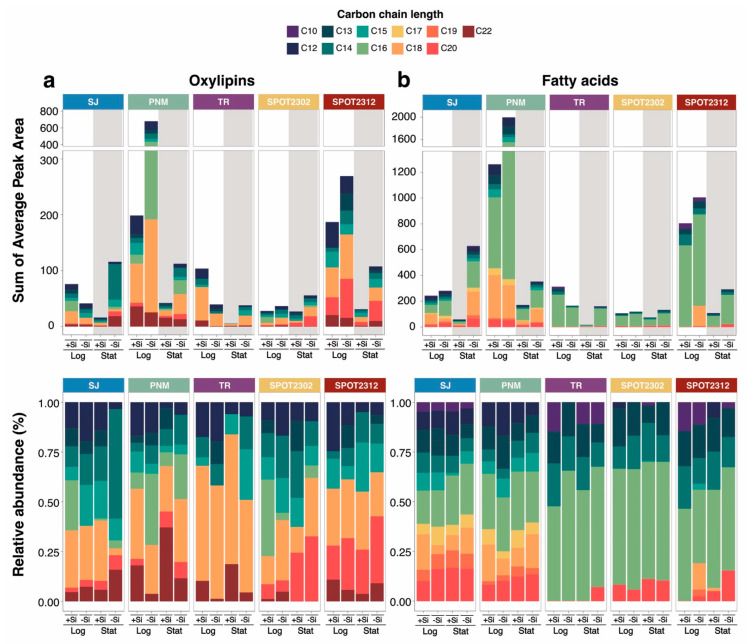
Overview of oxylipins and fatty acids by carbon chain length: (**a**) Sum of oxylipin peak areas for each feature (cell normalized, square root transformed, then averaged between triplicates) and their relative abundances; (**b**) sum of fatty acid peak areas for each feature (cell normalized, square root transformed, then averaged between triplicates) and their relative abundances. Peak areas for each feature are colored by carbon chain length. Diatom cultures collected during stationary growth are shaded in gray. Bar plots are separated by species and are denoted by the following acronyms: *Skeletonema japonicum* (SJ), *Pseudo-nitzschia multiseries* (PNM), *Thalassiosira rotula* (TR), SPOT2302 isolate (SPOT2302), and SPOT2312 isolate (SPOT2312). (+Si) denotes Si-replete cultures whereas (-Si) denotes Si-limited cultures. Log denotes cultures sampled during logarithmic growth whereas Stat denotes cultures sampled during stationary growth.

Si-limitation generally increased the release of oxylipins across all species, with the highest release per cell observed in *S. japonicum*, *P. multiseries*, and the SPOT2312 isolate (Figure 1). While the total amount of oxylipins differed by species and growth condition, a diverse set of carbon chain lengths were represented (Figure 1a and Figure S17). C14, C20, and C22 LOFAs were higher in *S. japonicum* during Si-limited, stationary growth. In *P. multiseries*, Si-limitation elevated the release of C12, C13, C15, 16, and C18 LOFAs during logarithmic growth. Interestingly, C16 and C20 LOFAs were not observed in *T. rotula* exudate despite previous observations of these LOFAs in studies investigating oxylipin biosynthesis [44,45]. In the SPOT2302 isolate, C20 LOFAs were particularly elevated with Si-limited, stationary growth. Lastly, in the SPOT2312 isolate, C12 and C18 LOFAs were higher during Si-limited, logarithmic growth similar to *P. multiseries*. The increased release of certain LOFAs with Si-limitation further supplements previous studies which observed higher oxylipin release with physiological stress. For instance, transgenic clones of *Phaeodactylum tricornutum* under oxidative stress released more C16, C18, C20, and C22 LOFAs compared to the wild-type [10]. Furthermore, stress induced by viral infection in *Chaetoceros sp*. also increased the release of C14, C16, C20, and C22 LOFAs [16]. Because many LOFAs from these chain lengths are known to be allelopathic [10,22,24,25], it is conceivable that diatoms like *S. japonicum* and *P. multiseries* could be better chemically defended under Si-limitation.

Additionally, Si-limitation increased the magnitude of fatty acids released though they were not as diverse compared to the oxylipins in terms of carbon chain length (Figure 1b). C16 fatty acids were abundant across all species and comprised most of the fatty acids in *T. rotula* and the SPOT isolates, which had a lower diversity of fatty acid by carbon chain-length. In contrast, *S. japonicum* and *P. multiseries* released an array of fatty acids ranging from C12–C16 and C17–C20 chain lengths (Figure 1b and Figure S18). Saturated C16 fatty acids were the largest component of the fatty acids (Figure 1b), and polyunsaturated fatty acids (PUFAs) were released more in Si-limited *S. japonicum*, *P. multiseries*, and the SPOT2312 isolate (Figures S19 and S20). The concomitant release of PUFAs with Si-limitation poses further implications for marine ecosystems, as PUFAs can act as energy sources that connect phytoplankton to other trophic levels. Because PUFAs like EPA are generally obtained from diet at higher trophic levels, increased release with Si-limitation could provide a source of energy for osmotrophic organisms and the trophic food webs they support. These results are consistent with a study that showed that DOM from Si-stressed diatoms promoted bacterial activity in culture better than DOM from nutrient replete cells [46].

Though it is generally understood that oxylipin biosynthesis terminating in PUA production begins right after cell membrane disruption, oxylipin release from intact cells can occur through mechanisms that are not fully understood [14,47]. Release of extracellular vesicles is one such mechanism, with the depletion of silicon in *Coscinodiscus radiatus* increasing the production of extracellular vesicles containing both oxylipins and fatty acids [48]. Therefore, we hypothesize extracellular vesicles could account for the increased release of LOFAs with Si-limitation in our study. While extracellular vesicles were not measured, future investigations profiling the production and lipidome of these vesicles could provide valuable insights on the mechanisms of oxylipin release from intact diatoms. Additionally, we hypothesize that the parallel increase in LOFAs and fatty acids with Si-limitation could also be attributed to cell “leakage” or the passive diffusion of intracellular material as DOM. Previous findings in Si-limited *S. marinoi* attributed the increase in PUAs like heptadienal and octadienal as a form of chemical defense to compensate for thinner siliceous cell walls [18]. Because the microstructures of diatom cell walls can affect diffusion [49], it is possible that altered cell wall morphology with Si-limitation (i.e., thinner frustules) could increase the leakage of intracellular LOFAs and fatty acids. Though silicon limitation in diatoms reduces cell wall silicification and compromises cell membranes [50,51,52], further observations regarding frustule thickness and membrane integrity are needed to validate our hypothesis of increased leakage with Si-limitation and explore the role vesicles and blebbing play in diatom ecology.

Lastly, we noted an absence of some PUFA precursors and LOFAs in *T. rotula* with Si-limitation (Figure 1 and Figure S21). Previous studies explored oxylipin production in *T. rotula* by stimulating grazing through freeze–thaw cycles or sonication, whereas we looked at the dissolved oxylipins after gently removing the cells from the media with filtration. Thus, it is possible that *T. rotula* might only produce oxylipins when grazed upon. *T. rotula* also has one of the lowest Si pmol per cell of any species that we included in our screening (Table 1), so it makes sense that its lipidome would respond less readily to Si-deplete media.

### 2.3. Differential Release of Oxylipins with Growth Phase

Intriguingly, we observed the highest release of LOFAs and fatty acids in *P. multiseries* and the SPOT2312 isolate during logarithmic growth (Figure 1a). These results oppose previous findings, which observed the highest oxylipin release with culture age [14,16,18,19,53]. Differences in our observations could be attributed to the species-specific release of LOFAs (Figures S22 and S23), which also display different trends based on carbon chain length. For instance, C20-derived compounds were both higher during stationary growth in *P. multiseries* and the SPOT isolates (Figure 1 and Figure S21). Trends within this group of compounds align with previous findings which observed higher release of 15-HpEPE and 15-HEPE during stationary growth in *Chaetoceros spp* and *Pseudo-nitzschia delicatissima* [16,53]. However, when considering the overall signal, trends in *P. multiseries* and the SPOT2312 were driven by C12 and C18 LOFAs, which were higher during logarithmic growth (Figure 1 and Figure S21).

The increased amount of LOFAs during logarithmic growth for *P. multiseries* and the SPOT2312 isolate is confounding, though it could be attributed to their potential role as chemical signals. For instance, oxylipins were observed to be inversely correlated with diatom abundance in phytoplankton assemblages in the Mediterranean [23]. Russo et al. proposed that this inverse relationship could be indicative of chemical signaling, with communication facilitated by stronger signals at low diatom abundances. Therefore, because C18 fatty acids are minor components in diatoms [54], it is possible that C18-derived LOFAs could serve as highly specific signals during low cell densities. While controlled experiments testing quorum sensing potential in diatoms have not been performed yet, the bacterium, *Pseudomonas aeruginosa*, and the fungi, *Aspergillus ochraceus*, display quorum sensing properties in response to oxylipins [55,56].

Furthermore, the increased release of LOFAs at lower cell densities could be attributed to the role of LOFAs as chemical defenses, allowing *P. multiseries* and SPOT2312 to proliferate during early stages of growth. It is possible that C18 LOFAs could facilitate interactions between diatoms and microbes during growth, allowing diatoms to “fine-tune” their microbiome as observed with other compounds [57]. PUAs, for instance, can have a differential effect on marine bacteria [13], stimulating or reducing growth of certain strains. Though oxylipin biosynthesis from C18 PUFAs has been documented in cyanobacteria, red algae, and fungi [58], their role as chemical signals in marine diatoms needs further elucidation.

### 2.4. Drivers of Species-Specific, Dissolved Lipidomes

**Figure 2:**
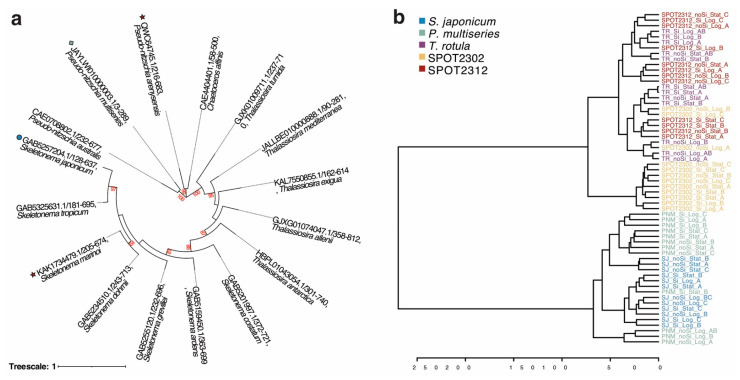
Analysis of diatom lipoxygenases and dissolved lipidomes: (**a**) phylogenetic analysis of lipoxygenase sequences aligned to the HMM profile of the lipoxygenase domain (PF00305); (**b**) dendrogram comparing annotated dissolved lipidomes using Euclidean distance and Ward clustering. NCBI accession numbers are displayed for each sequence. Bootstrap values are displayed in red. Reference lipoxygenase sequences are denoted by red stars while sequences from *Skeletonema japonicum* and *Pseudo-nitzschia multiseries* are denoted by a blue circle and a green diamond, respectively. For the dendrogram, samples are colored by species: *Skeletonema japonicum* (blue), *Pseudo-nitzschia multiseries* (green), *Thalassiosira rotula* (purple), SPOT2302 isolate (yellow), and SPOT2312 isolate (orange).

To bridge lipidomic analyses with the mechanisms behind oxylipin biosynthesis, we took a closer look into diatom lipoxygenases which are one of the first enzymes in oxylipin biosynthesis, inserting hydroperoxyl moieties on PUFAs [59,60]. Reference sequences annotated as lipoxygenases from *Pseudo-nitzschia arenysensis* (Accession number: QWC64745.1) and *Skeletonema marinoi* (Accession number: KAK1734479.1) were used to search for putative sequences from diatom genera in our study. Retrieved sequences were aligned to the lipoxygenase hidden Markov model (PF00305) prior to building a maximum likelihood tree (Figure 2a). Sequences formed four major groups based on the diatom genus: two *Pseudo-nitzschia* branches and a *Skeletonema* branch within the *Thalassiosira* branch. While more sequences from *Chaetoceros* are needed, it appears that lipoxygenase sequences from *C. affinis* are closely related to those of *P. arenysensis* while *P. australis* and *P. multiseries* share a different lipoxygenase. Furthermore, sequences from *Skeletonema* appear more closely related to sequences from *Thalassiosira* than those from *Pseudo-nitzschia*. Conversely, using a hierarchical clustering dendrogram, annotated lipidomes (n = 60 oxylipin and free fatty acids features) from *S. japonicum* were closely related to those of *P. multiseries* whereas those from *T. rotula* and the SPOT isolates formed another cluster (Figure 2b). This was further validated by multivariate analysis of the dissolved lipidomes using a partial least squares discriminant analysis (PLS-DA), which separated *S. japonicum* and *P. multiseries* from the other species alongside component 1 (33.1%, Figure S22). Considering only the LOFAs, a separate PLS-DA also clustered species in a similar manner, with C18 LOFAs having the highest variable importance in projection (VIP) scores (Figure S24). Remarkably, FFA 22:6 +3O had the highest VIP score across both PLS-DAs.

While most diatoms contain the molecular machinery for oxylipin biosynthesis [61], production of LOFAs and PUAs is not always conserved across species or even strains [62,63,64]. Previous bioinformatic investigations on the structure and function of lipoxygenases in diatoms attributed the variability in oxylipin products to small changes in amino acid residues, allowing diatoms to ‘fine-tune’ their lipoxygenases [65]. While our bioinformatic analysis did not include lipoxygenase sequences from other diatom genera, our results generally agree with previous findings on diatom lipoxygenases [65], with *Pseudo-nitzschia* sequences falling into two separate branches of the phylogenetic tree (Figure 2a). However, there is likely some other control on oxylipin production as *P. multiseries* and *S. japonicum* had the most distantly related lipoxygenase sequences but the most similar dissolved lipidomes (Figure 2b). Molecular insights into lipoxygenases from *Pseudo-nitzschia arenysensis* and *Fragilariopsis cylindrus* suggest localization differs between the two species, with lipoxygenases in *P. arenysensis* proposed to localize near chloroplasts and the endoplasmic reticulum [66,67]. Based on lipid compartmentalization, higher amounts of C18 and long chain PUFAs in the endoplasmic reticulum [68] could contribute to the C18 LOFA signature observed in the lipidomes of *S. japonicum* and *P. multiseries*. However, further subcellular studies are needed to determine where lipoxygenase localize across diatom genera.

**Figure 3:**
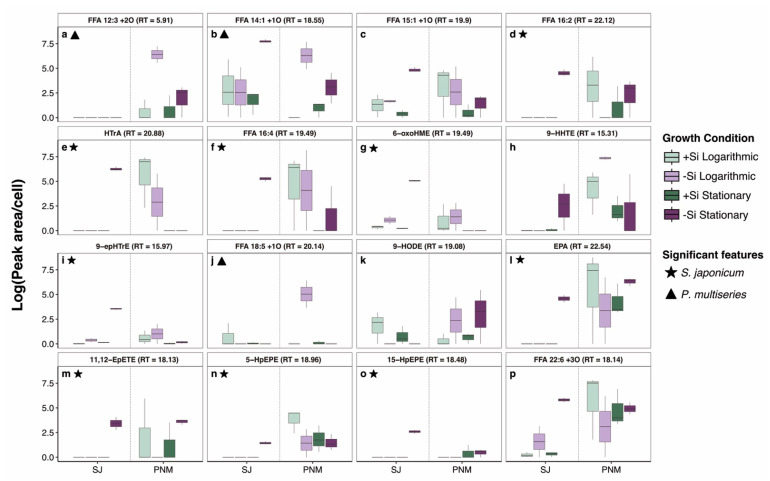
Boxplots of cell normalized and log transformed peak areas for features of interest in *Skeletonema japonicum* and *P. multiseries*: (**a**) FFA 12:3 +2O; (**b**) FFA 14:1 +1O; (**c**) FFA 15:1 +1O; (**d**) FFA 16:2; (**e**) hexadecatrienoic acid; (**f**) FFA 16:4; (**g**) 6-oxo-hexadecaenoic acid; (**h**) 9-hydroxy-hexadecatetraenoic acid; (**i**) 9-epoxy-hexadecatrienoic acid; (**j**) FFA 18:5 +1O; (**k**) 9-hydroxy-octadecadienoic acid; (**l**) Eicosapentaenoic acid; (**m**) 11,12-epoxy-eicosatetraenoic acid; (**n**) 5-hydroperoxy-eicosapentaenoic acid; (**o**) 15-hydroperoxy-eicosapentaenoic acid; (**p**) FFA 22:6 +3O. Si-replete samples are colored green whereas Si-limited samples are colored purple, with samples collected during logarithmic growth shaded lighter. Stars and triangles denote significant features from ANOVA in *S. japonicum* and *P. multiseries*, respectively.

Notably, while *S. japonicum* and *P. multiseries* had the most similar dissolved lipidomes, Si-limitation produced a distinct lipid signal with growth phase (Figure S22). Most of the significant features pulled out from the analysis of variance (Tukey HSD, *p*-value < 0.05) across *S. japonicum* were only higher during Si-limited, stationary growth (Table S1 and Figure S21). In this condition, C16 LOFAs (6-oxoHME, 9-epHTrE) and C20 LOFAs (11,12-epETE, 5-HpEPE, 15-HpEPE) were all elevated alongside many PUFA precursors (Figure 3). In *P. multiseries*, LOFAs like FFA 12:3 +2O, FFA 14:1 +1O, and FFA 18:5 +1O were significantly higher during Si-limited, logarithmic growth. Interestingly, C16 LOFAs and C16 PUFAs displayed opposing trends between the two species, with higher C16 LOFAs during Si-limited, logarithmic growth in *P. multiseries* (Figure 3). Differences with growth phase could be attributed to PUFA substrate availability as C16 PUFAs followed similar release trends. Because PUFA availability is controlled by lipases which liberate fatty acids from cell membranes [69], differed regulation of these enzymes in *S. japonicum* and *P. multiseries* could account for the variations observed in C16 PUFAs and LOFAs (Figure 3d–i).

### 2.5. Oxylipin Concentrations and Ecological Implications

**Figure 4:**
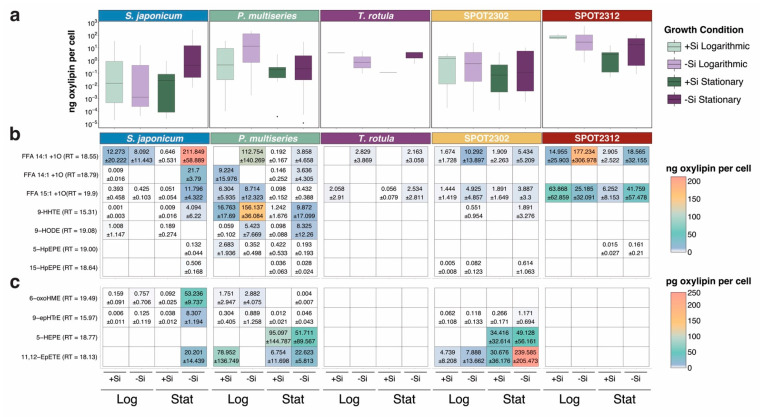
Estimated oxylipin quantities: (**a**) Sum of log-scaled, cell-normalized concentrations for MS2 confirmed oxylipins with representative functional groups in our authentic standard mix; (**b**) nanograms of oxylipin released per cell; (**c**) picograms of oxylipin released per cell. Calculated oxylipin concentrations were normalized to cell counts, then averaged across triplicates. ± denotes the standard deviation between triplicates. Features with no observed values are denoted by empty white boxes. Features are organized by carbon chain length. Samples are grouped by species and organized by culture condition.

To estimate ecologically relevant concentrations, linoleic acid oxylipin standards were used for the absolute quantification of MS2 confirmed oxylipins with represented functional groups (i.e., epoxy, hydroxy, etc.; Figure 4 and Figure S25). Calculated LOFA concentrations were particularly high in *S. japonicum* during Si-limited, stationary growth which totaled to 250.16 ng/cell, whereas LOFAs in *P. multiseries* totaled to 283.38 ng/cell during Si-limited, logarithmic growth. Of the C16 LOFAs, 9-HHTE had the highest concentration in both species followed by 6-oxoHME and 9-epHTrE. In contrast, in the Mediterranean, positional isomers of HEPE dominated the particulate LOFA signal throughout most of the year [23]. Concentrations of 5-HpEPE and 15-HpEPE were higher compared to 5-HEPE and 11,12-epETE. The C20 LOFA order of abundance roughly follows the order of proposed biosynthesis in diatoms with hydroperoxy acids being followed by hydroxy and epoxy acid production catalyzed by enzymes downstream of lipoxygenases.

Elevated oxylipin concentrations driven by Si-limitation in culture could extend to diatoms in Si-limited regions or during bloom demise when silicon becomes depleted [70]. As *Skeletonema* and *Pseudo-nitzschia* are common bloom-forming diatoms, elevated concentrations of LOFAs could impact various components of the marine ecosystem. Considering the estimation of 10,000 cells per liter of seawater, extrapolated concentrations of 9-HHTE could amount to 1.55 µM and 5.91 µM for *S. japonicum* and *P. multiseries*, respectively. For 11,12-epETE, extrapolated concentrations were lower at around 6.34 and 7.1 nM for *S. japonicum* and *P. multiseries*, respectively. Because these estimates are based on culture studies, it is important to note that concentrations for 9-HHTE and 11,12-epETE could be widely different in the environment. Proceeding forward with these assumptions, oxylipin concentrations in our study fall within the concentration range known to inhibit zooplankton grazing, phytoplankton competitors, and bacteria. For instance, Johnson et al. observed a 50% reduction in dinoflagellate grazing on *P. tricornutum* upon the addition of 1 nM of 15-hydroperoxy-eiocsatetraenoic acid (15-HpETE) [10]. Furthermore, particulate concentrations of LOFAs in the nM range have been observed to negatively impact copepod reproduction both at sea and in laboratory studies [22,24,25]. While the effect of LOFAs on other phytoplankton needs further evaluation, PUA concentrations higher than 1 µM were observed to decrease phytoplankton growth [8]. Similarly, more investigations are also required for bacteria. Though ~3 nM of 15-HEPE has been observed to reduce the growth of the bacterium *K. algicida* [14], incubations with natural populations from sinking particles observed higher enzyme activity with the addition of 1 µM 15-HpETE [71]. Thus, LOFAs might act similarly to PUAs, allowing oxylipin-resistant microbes to outcompete oxylipin-sensitive microbes on sinking particles and increase the remineralization of POM [13,72].

## 3. Conclusions and Broader Impacts

Oxylipin production, mainly PUAs, occurs when diatoms are stressed or wounded by grazing [17,18,19,22,71]. Here, we present evidence that physiological changes associated with Si-limitation and growth phase elicit the extracellular release of LOFAs across five diatom species. Our results suggest that the release of LOFAs in each growth phase varies by diatom, with Si-deplete conditions generally increasing the quantities released. The dissolved lipidomes of *S. japonicum* and *P. multiseries* responded most distinctly to Si-limitation, with concentrations as high as 250.16 and 283.38 ng/cell, respectively.

Ultimately, the increase in LOFAs during Si-limitation has important implications for understanding the fate of diatom blooms. These compounds have previously been shown to play roles in deterring or inhibiting grazing activity and growth rate of protist grazers [10,26,73] and disrupting the development of copepod eggs and nauplii [9,11,25]. Without accounting for viral losses, these findings raise the possibility that during Si-limitation, diatom bloom biomass may persist longer due to oxylipin depressed grazing. Furthermore, our systematic study of lipidome plasticity across diatom species generated new hypotheses regarding mechanisms for the organic matter release during Si stress, subcellular determinants of oxylipin diversity, and the use of specific oxylipins as chemical signaling during balanced growth.

## 4. Materials and Methods

### 4.1. Diatom Cultures

*Skeletonema japonicum* (EBL-01) and *Pseudo-nitzschia multiseries* (EBL-27) were isolated in 2021 from Monterey Bay and kindly provided by Holly Bowers. *Thalassiosira rotula* (CCMP3362) was obtained from Bigelow National Center for Marine Algae and microbiota (NCMA), and cultures SPOT2302 (unidentified diatom) and SPOT2312 (*Chaetoceros* sp.) were isolated in 2023 from the San Pedro Ocean Time Series by Matt Johnson. All stock cultures were maintained at 15 °C in 35 PSU coastal sea water from Martha’s Vineyard Sound amended with f/2 + Si media. For experiments herein, diatom cultures were grown in triplicate batch cultures under either nutrient replete (f/2 + Si) or silicon-limited (f/2 − Si) conditions, and cells were harvested at both logarithmic and stationary phase of growth. In our study, stationary growth was defined as cultures sampled during the final sampling point, which displayed lower growth rates compared to the cultures sampled earlier defined as logarithmic growth. Growth rates were calculated via daily cell counts using microscopy (Table S2).

### 4.2. Lipid Extraction and Data Acquisition

Media (~15 mL) collected from cultures was filtered through a 0.2 µm membrane filter (Durapore, Sigma-Aldrich, St. Louis, MO, USA) and loaded onto 6 cc HLB-SPE cartridges containing 150 mg of sorbent (Oasis HLB, Waters, Milford, MA, USA). Before loading culture media, cartridges were conditioned with ~5 mL of methanol (Optima LC/MS grade, ThermoFisher Scientific, Waltham, MA, USA) then with ~5 mL of water (Optima LC/MS grade, ThermoFisher Scientific). Cartridges were stored at −80 °C. Before extraction, cartridges were further rinsed with water (LC/MS grade). Dissolved lipids were then eluted with 2 mL of methanol (LC/MS grade) into combusted vials containing butylated hydroxytoluene (antioxidant) and 10 µL of deuterated internal standard (EquiSPLASH LIPIDOMIX, Avanti Polar Lipids, Alabaster, AL, USA; diluted to 50 ug/mL per compound). Extracts were transferred into combusted HPLC vials, capped under nitrogen gas, and stored at −80 °C. Samples were injected at a volume of 2 µL and separated on a C8 column (155 mm × 2.1 mm × 2.6 μm) using reverse phase ultra-high-pressure liquid chromatography (Vanquish, ThermoFisher Scientific). A solvent gradient of 45A/55B to 1A/99B over 30 min was used. Eluent A consisted of LC-grade water and Eluent B consisted of acetonitrile and isopropanol (70:30), both of which contained 10 mM ammonium acetate and 0.1% acetic acid. Mass spectrometric analysis was performed in negative mode (2500 V source energy) using a high-resolution accurate-mass spectrometer (Orbitrap ID-X, ThermoFisher Scientific) in data dependent acquisition mode with an AGC target of 5E-4 at a resolution of 120,000 and a mass inclusion list for fragmentation of [M−H]^−^ adducts of known diatom oxylipins. Stepped collision energy (25, 30, 35%) was used for MS2 fragmentation before being routed back to the orbitrap mass analyzer.

### 4.3. Lipidomic Analysis

Raw files were converted to .mzXML format using msconvert [74]. Feature annotation was performed using the LOBSTAHS R package (R version 4.2.2) which utilized XCMS and CAMERA to align chromatograms and integrate peaks [42,75,76]. A total of 5852 features were identified and a subset was annotated as free fatty acids (FFA), polyunsaturated aldehydes (PUA), intact monoacylglycerols (IP-MAG), and intact diacylglycerols (IP-DAG). Annotations as FFAs and PUAs (n = 256) were retained for manual verification in MAVEN [77], resulting in 60 features with high-quality extracted ion chromatograms. Peak areas were blank subtracted, then normalized to an internal standard, 1-oleoyl(d7)-2-hydroxy-sn-glycero-3-phosphoethanolamine (LPE), and cell counts. Because many of these features lacked MS2 fragmentation, a subset of samples was run again with a given list of *m*/*z* ratios to fragment. A list of *m*/*z* ratios was retrieved from the initial LOBSTAHS analysis. To confirm annotations, raw files from both runs were converted to .ABF format using (Reifycs Abf Converter) and analyzed separately in MSDIAL [43]. Features with MS2 fragmentation were confirmed using modeled fragmentation databases via Competitive Fragmentation Modeling-ID (CFMID) and an ESI(-)-MS/MS MSDIAL database from authentic standards. Annotations from LOBSTAHS (*n* = 60) and MSDIAL were matched based on observed *m*/*z* and retention time similarity (threshold: 0.001) and retention time (threshold: 0.1). Differences in retention time were observed from the first run, so a threshold of 0.25 min was applied to match features from the second run. For compounds with MS2 verification, concentrations were estimated from peak area using a linoleic acid oxylipin standard curve (Linoleic Acid Oxylipins MaxSpec^®^ LC-MS Mixture, Cayman Chemical, Ann Arbor, MI, USA). Estimated concentrations were then normalized to cell counts.

### 4.4. Statistical Analysis

Statistical analyses were performed in the online version of MetaboAnalyst 5.0 [78], using peak areas (cell normalized and log-transformed) generated from the LOBSTAHS workflow. Partial Least Squares Discriminant Analysis (PLSDA) was performed on all samples and verified via cross validation and permutation tests. Principal component analysis (PCA) and analysis of variance (ANOVA) were performed on samples from each species separately. The dendrogram comparing lipidomic samples was constructed using Euclidean distance and a Ward clustering algorithm.

### 4.5. Lipoxygenase Sequence Analysis

Reference sequences annotated as lipoxygenases from *Pseudo-nitzschia arenysensis* (QWC64745.1) and *Skeletonema marinoi* (KAK1734479.1) were used to search for putative lipoxygenase sequences via the Basic Local Alignment Search Tool (BLAST) from the National Center of Biotechnology Information (NCBI). Protein sequences were searched for in the non-redundant protein sequences (nr) and the transcriptome shotgun assembly proteins (tsa_nr) databases. Nucleotide sequences were searched for using translated BLAST in the whole-genome shotgun contigs (wgs) and transcriptome shotgun assembly (TSA) databases. For nucleotide sequences, InterPro Scan was used to identify lipoxygenase domains and translate sequences. Curated protein sequences were then aligned to the lipoxygenase hidden Markov model, PF00305, obtained from InterPro. For alignment, an E-value threshold of 1E-5 was used to construct a maximum likelihood tree via RAxML. Tree visualization was performed in iTOL. The following sequences were used: *Skeletonema japonicum* (GAB5257204.1), *Skeletonema ardens* (GAB5159450.1), *Skeletonema costatum* (GAB5201997.1), *Skeletonema dohrnii* (GAB5234510.1), *Skeletonema grevillei* (GAB5255120.1), *Skeletonema tropicum* (GAB5325631.1), *Chaetoceros affinis* (CAE4404401.1), *Pseudo-nitzschia australis* (CAE0708802.1), *Pseudo-nitzschia multiseries* (JAYLWI010000003.1), *Thalassiosira exigua* (KAL7550855.1), *Thalassiosira allenii* (GJXG01074047.1), *Thalassiosira antarctica* (HBPL01043054.1), *Thalassiosira tumida* (GJXK01009711.1), and *Thalassiosira mediterranea* (JALLBE010000888.1).

## Figures and Tables

**Table 1:** Diatom species and isolates cultured in this study. Reports of cell length, silicon content, Si:C ratio, and distribution were obtained from other studies.

Diatom	Label	Morphology	Cell Length (µm)	Silicon Content (pmol/cell)	Si:C	Distribution	References
*Skeletonema japonicum*	SJ	Centric	5.3–5.5	0.03–3.40 ^a^	0.07–0.11 ^a^	Cold temperate coasts and upwelling zones	[27,28,29,30]
*Pseudo-nitzschia multiseries*	PNM	Pennate	50–140	1.07, 1.9 ^b^	0.16, 0.21	Coasts alongside the NW Pacific, NE Atlantic, and Mediterranean	[31,32,33,34,35,36]
*Thalassiosira rotula*	TR	Centric	10.5–65.8	0.07 ^b^, 0.09 ^b^	0.09	North Pacific and Atlantic, Mediterranean, Southern Ocean	[37,38,39,40]
*Chaetoceros* sp.	SPOT2312	Centric		0.12–7.30 ^c^	0.09–0.15 ^c^	Global	[37,41]
Unidentified	SPOT2302	Centric					

^a^ Reported values for *Skeletonema costatum.* ^b^ Values converted from pg Si/cell to pmol Si/cell. ^c^ Range across *Chaetoceros* species and strains.

**Table 2:** Oxylipins within the diatom dissolved lipidome that were annotated via LOBSTAHS and MSDIAL. Compounds identified in LOBSTAHs with confirmed MS2 fragmentation in MSDIAL were matched based on mass to charge ratio and observed retention time, and given a more detailed annotation with structural and positional information in addition to the simple elemental information on carbon number, double bond, and extra oxygens provided by LOBSTAHS.

Compound	Acronym	LOBSTAHS Annotation	Matched Fragments	LOBSTAHS (*m*/*z*)	MSDIAL (*m*/*z*)	LOBSTAHS Retention Time (mins)	MSDIAL Retention Time (mins)
		FFA 12:2 +1O		211.13397	211.13397	14.90	14.94
		FFA 12:2 +1O		211.13398	211.13403	14.56	14.55
		FFA 12:3 +2O		225.11320	225.11342	7.88	7.87
		FFA 12:3 +2O		225.11324	225.11313	5.91	5.90
		FFA 13:2 +1O		225.14962	225.14963	13.21	13.25
		FFA 13:2 +2O		241.14466	241.14436	11.35	11.32
9-hydroxy-tetradeca-7-enoic acid		FFA 14:1 +1O	4	241.18095	241.18069	18.55	18.37
9-hydroxy-tetradeca-7-enoic acid		FFA 14:1 +1O	3	241.18095	241.18074	18.79	18.86
		FFA 14:1 +1O		241.18097	241.18097	17.89	17.85
		FFA 15:2 +1O		253.18091	253.18076	15.73	15.74
		FFA 15:1 +1O		255.19631	255.19649	16.76	16.80
9-hydroxy-pentadeca-10-enoic acid		FFA 15:1 +1O	3	255.19649	255.19685	19.90	19.74
		FFA 15:1 +1O		255.19668	255.19658	20.10	20.12
		FFA 15:1 +1O		255.19674	255.19664	19.28	19.31
9-hydroxy-hexadecatetraenoic acid	9-HHTE	FFA 16:4 +1O	3	263.16516	263.16525	15.31	15.46
		FFA 18:5 +1O		289.18101	289.18088	20.14	20.15
		FFA 18:4 +1O		291.19644	291.19653	19.93	19.91
		FFA 18:4 +1O		291.19675	291.19672	21.77	21.77
		FFA 18:2 +1O		295.22768	295.22784	20.61	20.56
		FFA 18:2 +1O		295.22769	295.22751	20.31	20.29
9-hydroxy-octadecadienoic acid	9-HODE	FFA 18:2 +1O	3	295.22776	295.22739	19.08	19.06
		FFA 18:1 +1O		297.24343	297.24298	21.79	21.77
11,12-epoxy-eicosatetraenoic acid	11,12-EpETE	FFA 20:5 +1O	6	317.21215	317.21219	18.13	18.30
6-ketoprostaglandin E1	6-keto-PGE1	FFA 20:4 +4O	3	367.21191	367.21179	12.90	12.90
		FFA 22:6 +3O		375.21731	375.21729	18.14	18.17
		FFA 22:4 +4O		395.24372	395.24326	15.17	15.19
		FFA 16:1 +1O		269.21213		18.69	
		FFA 18:3 +3O		325.20192		13.40	
9-epoxy-hexadecatrienoic acid	9-epHTrE		6		263.16525		15.97
6-oxo-hexadecaenoic acid	6-oxoHME		5		267.19632		19.49
15-deoxyprostaglandin D2	15-deoxy-PGD2		3		333.20682		15.74
15-hydroperoxy-eicosapentaenoic acid	15-HpEPE		18		315.19614		18.48
5-hydroperoxy-eicosapentaenoic acid	5-HpEPE		10		315.1965		18.96
5-hydroxy-eicosapentaenoic acid	5-HEPE		6		317.21188		18.78

## Data Availability

The files from the lipidomic analysis have been deposited into the MassIVE repository; see https://doi.org/doi:10.25345/C54X54W08.

## Supplementary Materials

The following supporting information can be downloaded at https://www.mdpi.com/article/10.3390/md23110424/s1: Figure S1: Fragmentation of 9-hydroxy-tetradeca-10-enoic acid, Figure S2: Fragmentation of 9-hydroxy-tetradeca-10-enoic acid, Figure S3: Fragmentation of 9-hydroxy-pentadeca-10-enoic acid, Figure S4: Fragmentation of 9-hydroxy-hexadecatetraenoic acid, Figure S5: Fragmentation of 9-hydroxy-octadecadienoic acid, Figure S6: Fragmentation of 11,12-epoxy-eicosatetraenoic acid, Figure S7: Fragmentation of 6-ketoprostaglandin E1, Figure S8: Fragmentation of 9-epoxy-hexadecatrienoic acid, Figure S9: Fragmentation of 6-oxo-hexadecaenoic acid, Figure S10: Fragmentation of 15-deoxyprostaglandin D2, Figure S11: Fragmentation of 15-hydroperoxy-eicosapentaenoic acid, Figure S12: Fragmentation of 5-hydroperoxy-eicosapentaenoic acid, Figure S13: Fragmentation of 5-hydroxy-eicosapentaenoic acid, Figure S14: Fragmentation of Hexadecatrienoic acid. Figure S15: Fragmentation of Stearic acid. Figure S16: Fragmentation of Eicosapentaenoic acid, Figure S17: Boxplots of oxylipin peak areas by carbon chain length, Figure S18: Boxplots of fatty acid peak areas by carbon chain length, Figure S19: Boxplots of oxylipin peak areas by degree of saturation, Figure S20: Degree of saturation for oxylipins and fatty acids, Figure S21: Cell normalized and log transformed peak area for each feature, Figure S22: Score plots from PLSDA and PCA on all features annotated by LOBSTAHS, Figure S23: Comparison between PCA and PLSDA, Figure S24: PLSDA on features annotated as LOFAs. Figure S25: Linoleic acid oxylipin standard curves, Table S1: Significant compounds from ANOVA, Table S2: Growth rates for diatom species and culture condition.

## Abbreviations

The following abbreviations are used in this manuscript:

Si-limitation	Silicon limitation
POM	Particulate organic matter
DOM	Dissolved organic matter
TEP	Transparent exopolymer particles
PUA	Polyunsaturated aldehyde
LOFA	Linear oxygenated fatty acid
SPOT	San Pedro Ocean Time Series
6-oxoHME	6-oxo-hexadecaenoic acid
9-EpHTrE	9-epoxy-hexadecatrienoic acid
9-HHTE	9-hydroxy-hexadecatetraenoic acid
9-HODE	9-hydroxy-octadecadienoic acid
5-HpEPE	5-hydroperoxy-eicosapentaenoic acid
15-HpEPE	15-hydroperoxy-eicosapentaenoic acid
5-HEPE	5-hydroxy-eicosapentaenoic acid
11,12-EpETE	11,12-epoxy-eicosatetraenoic acid
15-HpETE	15-hydroperoxy-eiocsatetraenoic acid
6-keto-PGE1	6-ketoprostaglandin E1
15-deoxy-PGD2	15-deoxyprostaglandin D2
HTrA	Hexadecatrienoic acid
EPA	Eicosapentaenoic acid
PLSDA	Partial Least Squares Discriminant Analysis
PCA	Principal Component Analysis
FFA	Free fatty acid
ANOVA	Analysis of variance
PUFA	Polyunsaturated fatty acids
