# Supplementary material for: Lipidomic Screening of Marine Diatoms Reveals Release of Dissolved Oxylipins Associated with Silicon Limitation and Growth Phase
Source: Mar Drugs. 2025 Oct 31;23(11):424. doi: 10.3390/md23110424 (PMC12653812; doi:10.3390/md23110424)
Supplement: Supplementary file 1 [file marinedrugs-23-00424-s001.zip › marinedrugs_3931309_supplementary.pdf]

Supplementary Tables and Figures

Table S1: Significant compounds from ANOVA.

LOBSTAHS Annotation	f value	p value	Log (p value)	FDR	Tukey's HSD
FFA 16:3_mz_249.18615_RT_20.88	245.02	3.29E-08	7.4825	1.19E-06	SJ_noSi_Stat-SJ_noSi_Log; SJ_Si_Log-SJ_noSi_Stat; SJ_Si_Stat-SJ_noSi_Stat
FFA 14:1 +1O_mz_241.18095_RT_18.79	231.62	4.11E-08	7.386	1.19E-06	SJ_noSi_Stat-SJ_noSi_Log; SJ_Si_Log-SJ_noSi_Stat; SJ_Si_Stat-SJ_noSi_Stat
FFA 16:4_mz_247.17051_RT_19.49	201.23	7.16E-08	7.145	1.38E-06	SJ_noSi_Stat-SJ_noSi_Log; SJ_Si_Log-SJ_noSi_Stat; SJ_Si_Stat-SJ_noSi_Stat
FFA 16:2_mz_251.20166_RT_22.12	99.365	1.14E-06	5.9445	1.65E-05	SJ_noSi_Stat-SJ_noSi_Log; SJ_Si_Log-SJ_noSi_Stat; SJ_Si_Stat-SJ_noSi_Stat
FFA 20:5_mz_301.2172_RT_22.54	88.084	1.81E-06	5.7416	2.10E-05	SJ_noSi_Stat-SJ_noSi_Log; SJ_Si_Log-SJ_noSi_Stat; SJ_Si_Stat-SJ_noSi_Stat
FFA 20:5 +1O_mz_317.21215_RT_18.13	34.707	6.19E-05	4.2081	0.00059872	SJ_noSi_Stat-SJ_noSi_Log; SJ_Si_Log-SJ_noSi_Stat; SJ_Si_Stat-SJ_noSi_Stat
FFA 18:2 +1O_mz_295.22768_RT_20.61	12.544	0.0021554	2.6665	0.017859	SJ_noSi_Stat-SJ_noSi_Log; SJ_Si_Log-SJ_noSi_Log; SJ_Si_Stat-SJ_noSi_Log
FFA 15:0_mz_241.21715_RT_24	11.911	0.0025457	2.5942	0.018457	SJ_Si_Stat-SJ_noSi_Log; SJ_Si_Stat-SJ_noSi_Stat; SJ_Si_Stat-SJ_Si_Log
FFA 18:0_mz_283.26395_RT_26.57	8.3675	0.0075376	2.1228	0.048575	SJ_noSi_Stat-SJ_noSi_Log; SJ_Si_Stat-SJ_noSi_Stat
15-HpEPE	427.8	3.62E-09	8.4419	1.81E-08	SJ_noSi_Stat-SJ_noSi_Log; SJ_Si_Log-SJ_noSi_Stat; SJ_Si_Stat-SJ_noSi_Stat
6-oxoHME	95.683	1.32E-06	5.8809	2.58E-06	SJ_noSi_Stat-SJ_noSi_Log; SJ_Si_Log-SJ_noSi_Log; SJ_Si_Stat-SJ_noSi_Log; SJ_Si_Log-SJ_noSi_Stat; SJ_Si_Stat-SJ_noSi_Stat
5-HpEPE	91.76	1.55E-06	5.8104	2.58E-06	SJ_noSi_Stat-SJ_noSi_Log; SJ_Si_Log-SJ_noSi_Stat; SJ_Si_Stat-SJ_noSi_Stat
9-epHTrE	24.411	0.00022216	3.6533	0.0002777	SJ_noSi_Stat-SJ_noSi_Log; SJ_Si_Log-SJ_noSi_Stat; SJ_Si_Stat-SJ_noSi_Stat
FFA 18:5 +1O_mz_289.18101_RT_20.14	58.945	8.49E-06	5.0713	0.00049216	PNM_noSi_Log - PNM_noSi_Stat; PNM_noSi_Log - PNM_Si_Log; PNM_noSi_Log - PNM_Si_Stat

FFA 20:0_mz_311.29556_RT_27.45	27.066	0.00015341	3.8142	0.0044488	PNM_noSi_Log - PNM_noSi_Stat; PNM_noSi_Log - PNM_Si_Log; PNM_noSi_Log - PNM_Si_Stat; PNM_noSi_Stat - PNM_Si_Log; PNM_noSi_Stat - PNM_Si_Stat
FFA 13:2_mz_209.15467_RT_15.07	23.99	0.00023637	3.6264	0.0045699	PNM_noSi_Log - PNM_noSi_Stat; PNM_noSi_Log - PNM_Si_Stat; PNM_Si_Log - PNM_noSi_Stat; PNM_Si_Log - PNM_Si_Stat
FFA 14:1 +1O_mz_241.18095_RT_18.55	20.529	0.0004096	3.3876	0.0059392	PNM_noSi_Log - PNM_noSi_Stat; PNM_noSi_Log - PNM_Si_Log; PNM_noSi_Log - PNM_Si_Stat; PNM_noSi_Stat - PNM_Si_Log; PNM_noSi_Stat - PNM_Si_Stat
FFA 12:3 +2O_mz_225.11324_RT_5.91	16.605	0.00085079	3.0702	0.0098692	PNM_noSi_Log - PNM_noSi_Stat; PNM_noSi_Log - PNM_Si_Log; PNM_noSi_Log - PNM_Si_Stat
FFA 12:3 +2O_mz_225.11324_RT_5.91	682.22	5.65E-10	9.2482	1.30E-08	TR_noSi_Stat-TR_noSi_Log; TR_Si_Log-TR_noSi_Log; TR_Si_Stat-TR_noSi_Log
FFA 10:3_mz_165.09213_RT_11.59	51.962	1.37E-05	4.8634	0.00015752	TR_noSi_Stat-TR_noSi_Log; TR_Si_Log-TR_noSi_Log; TR_Si_Stat-TR_noSi_Log; TR_Si_Stat-TR_noSi_Stat; TR_Si_Stat-TR_Si_Log
FFA 16:0_mz_255.23282_RT_24.98	11.879	0.0025677	2.5905	0.019686	TR_Si_Stat-TR_noSi_Log; TR_Si_Stat-TR_noSi_Stat; TR_Si_Stat-TR_Si_Log
FFA 18:3 +3O_mz_325.20192_RT_13.4	8.673	0.0067796	2.1688	0.036107	TR_Si_Stat-TR_Si_Log
FFA 16:4_mz_247.17041_RT_17.87	8.2528	0.0078495	2.1052	0.036107	TR_Si_Stat-TR_Si_Log
FFA 13:2_mz_209.15433_RT_11.8	7.61	0.0099287	2.0031	0.03806	TR_Si_Stat-TR_Si_Log
FFA 18:1 +1O_mz_297.24343_RT_21.79	6.9169	0.013002	1.886	0.042721	TR_Si_Stat-TR_Si_Log
FFA 16:1_mz_253.21747_RT_23.66	18.393	0.00059967	3.2221	0.01799	SPOT02_Si_Log-SPOT02_noSi_Log; SPOT02_Si_Log-SPOT02_noSi_Stat; SPOT02_Si_Stat-SPOT02_Si_Log
FFA 20:5_mz_301.2172_RT_22.54	36.781	5.00E-05	4.3012	0.0016494	SPOT12_noSi_Stat-SPOT12_noSi_Log; SPOT12_Si_Log-SPOT12_noSi_Stat; SPOT12_Si_Stat-SPOT12_noSi_Stat
FFA 10:3_mz_165.09213_RT_11.59	17.536	0.00070643	3.1509	0.011656	SPOT12_noSi_Stat-SPOT12_noSi_Log; SPOT12_Si_Stat-SPOT12_noSi_Log; SPOT12_Si_Log-SPOT12_noSi_Stat; SPOT12_Si_Stat-SPOT12_Si_Log
FFA 12:3 +2O_mz_225.1132_RT_7.88	11.257	0.0030464	2.5162	0.03351	SPOT12_Si_Stat-SPOT12_noSi_Log; SPOT12_Si_Stat-SPOT12_Si_Log

Table S2: Growth rates for diatom species and culture condition.

Species	Growth phase	Condition	Average Growth Rate
S. japonicum	Logarithmic	+Si	1.068 ± 0.067
	Logarithmic	-Si	0.644 ± 0.199
	Stationary	+Si	0.184 ± 0.113
	Stationary	-Si	-0.181 ± 0.079
P. multiseries	Logarithmic	+Si	0.773 ± 0.201
	Logarithmic	-Si	0.513 ± 0.18
	Stationary	+Si	0.563 ± 0.048
	Stationary	-Si	0.174 ± 0.102
T. rotula	Logarithmic	+Si	0.256 ± 0.022
	Logarithmic	-Si	0.241 ± 0.074
	Stationary	+Si	0.125 ± 0.044
	Stationary	-Si	-0.122 ± 0.024
SPOT2302	Logarithmic	+Si	1.257 ± 0.052
	Logarithmic	-Si	1.109 ± 0.039
	Stationary	+Si	-0.229 ± 0.073
	Stationary	-Si	-0.047 ± 0.156
SPOT2312	Logarithmic	+Si	Few cells
	Logarithmic	-Si	Few cells
	Stationary	+Si	0.597 ± 0.105
	Stationary	-Si	0.158 ± 0.125

a

LOBSTAHS Annotation	FFA 14:1 +1O (m/z = 241.1809456, RT = 18.55)
MSDIAL Annotation	FA 14:1 +1O_mz_241.18069_RT_18.367
Ref. SMILES	OC(=O)CCCCCCCC(O)C=CCCC

b

Sample m/z	Fragment Abundance	Reference m/z	Fragment Abundance	m/z difference
59.01346	3153	59.01385117	12.25	0.00039
195.17374	64531	195.1754456	7.239999771	0.00171
223.16977	6253	223.1703491	33.43999863	0.00058
241.18042	244999	241.1809235	100	0.00050

c

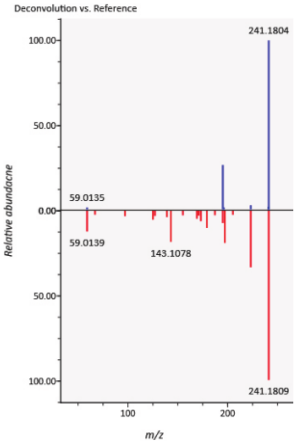

d

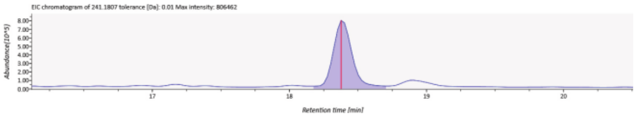

e

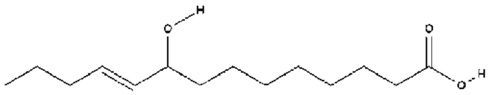

Figure S1: Fragmentation of 9-hydroxy-tetradeca-10-enoic acid. (a) LOBSTAHS and MSDIAL annotation, (b) matched fragments, (c) sample fragmentation (blue) against reference fragmentation (red), (d) EIC, and (e) compound structure.

a

LOBSTAHS Annotation	FFA 14:1 +1O (m/z = 241.1809488, RT = 18.79)
MSDIAL Annotation	FA 14:1 +1O_mz_241.18074_RT_18.861
Ref. SMILES	OC(=O)CCCCCCCC(O)C=CCCC

b

Sample m/z	Fragment Abundance	Reference m/z	Fragment Abundance	m/z difference
59.00763	2768	59.01385117	12.25	0.006221166
197.18837	6435	197.1910858	18.98999977	0.002715815
241.17639	9706	241.1809235	100	0.004533462

c

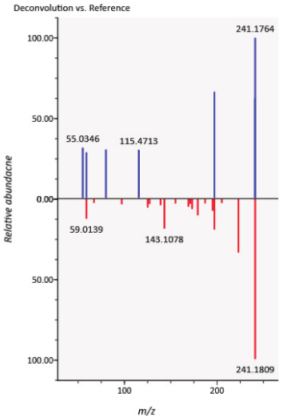

d

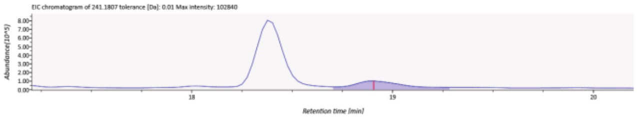

e

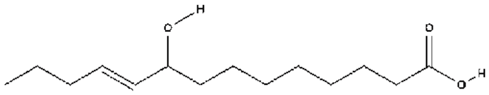

Figure S2: Fragmentation of 9-hydroxy-tetradeca-10-enoic acid. (a) LOBSTAHS and MSDIAL annotation, (b) matched fragments, (c) sample fragmentation (blue) against reference fragmentation (red), (d) EIC, and (e) compound structure.

a

**LOBSTAHS Annotation**	FFA 15:1 +1O (m/z = 255.1964904, RT = 19.9)
**MSDIAL Annotation**	FA 15:1 +1O_mz_255.19685_RT_19.736
**Ref. SMILES**	OC(=O)CCCCCCCC(O)C=CCCCC

b

Sample m/z	Fragment Abundance	Reference m/z	Fragment Abundance	m/z difference
58.9692	2783	59.01385117	10.92000008	0.044651166
209.1927	5095	209.1910858	7.769999981	0.001614185
255.19652	13410	255.1965637	100	4.37207E-05

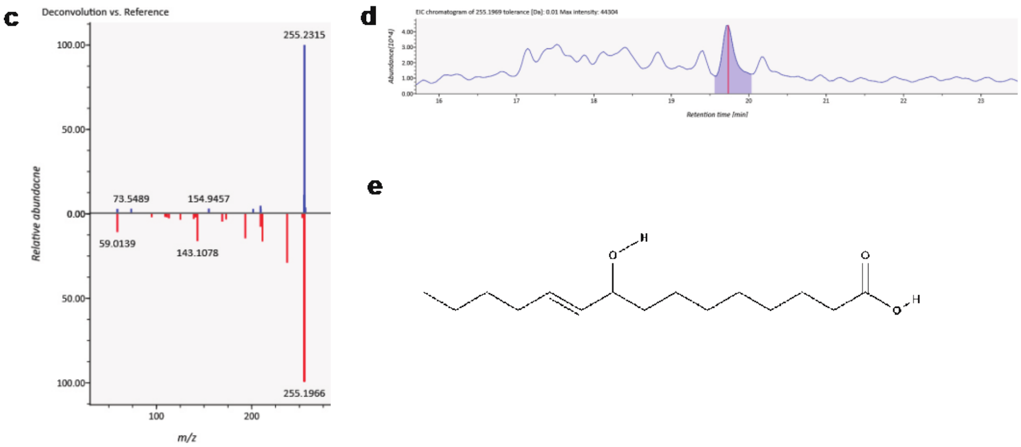

Figure S3: Fragmentation of 9-hydroxy-pentadeca-10-enoic acid. (a) LOBSTAHS and MSDIAL annotation, (b) matched fragments, (c) sample fragmentation (blue) against reference fragmentation (red), (d) EIC, and (e) compound structure.

a

**LOBSTAHS Annotation**	FFA 16:4 +1O (m/z = 263.1651597, RT = 15.31)
**MSDIAL Annotation**	9-HHTE_mz_263.16525_RT_15.463
**Ref. SMILES**	C=CCC=CC=CC(O)CC=CCCCCC(=O)[O-]

b

Sample m/z	Fragment Abundance	Reference m/z	Fragment Abundance	m/z difference
59.0133	32714	59.01385117	31.16439438	0.000551166
219.17584	2780	219.1754456	18.02619362	0.000394443
263.16306	23367	263.1652832	76.87561798	0.002223203

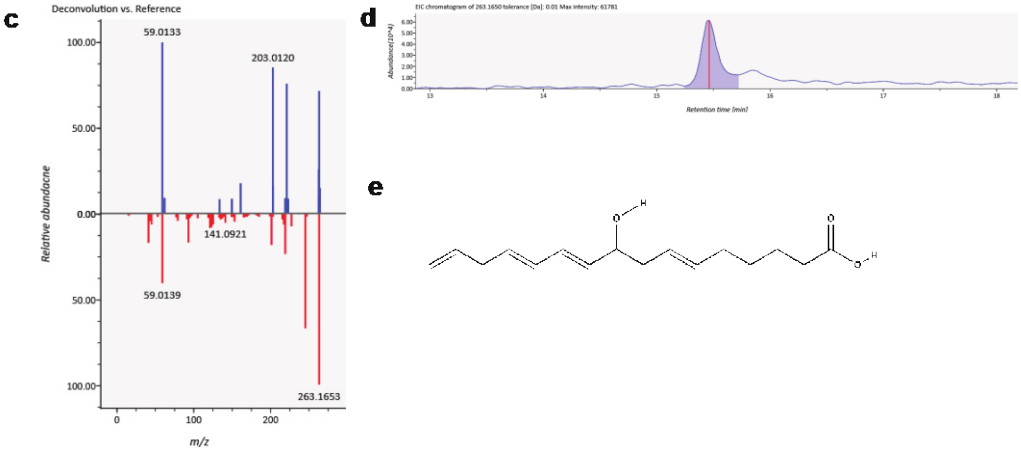

Figure S4: Fragmentation of 9-hydroxy-hexadecatetraenoic acid. (a) LOBSTAHS and MSDIAL annotation, (b) matched fragments, (c) sample fragmentation (blue) against reference fragmentation (red), (d) EIC, and (e) compound structure.

a

LOBSTAHS Annotation	FFA 18:2 +1O (m/z = 295.227761, RT = 19.08)
MSDIAL Annotation	9-HODE_mz_295.22739_RT_19.06
Ref. SMILES	CCCCC/C=C\C=C/C(O)CCCCCCCC(=O)O

b

Sample m/z	Fragment Abundance	Reference m/z	Fragment Abundance	m/z difference
171.10165	2863	171.1130066	116141936	0.011356592
277.21631	6465	277.240448	142094816	0.024137998
295.22733	4418	295.2550049	79244592	0.027674883

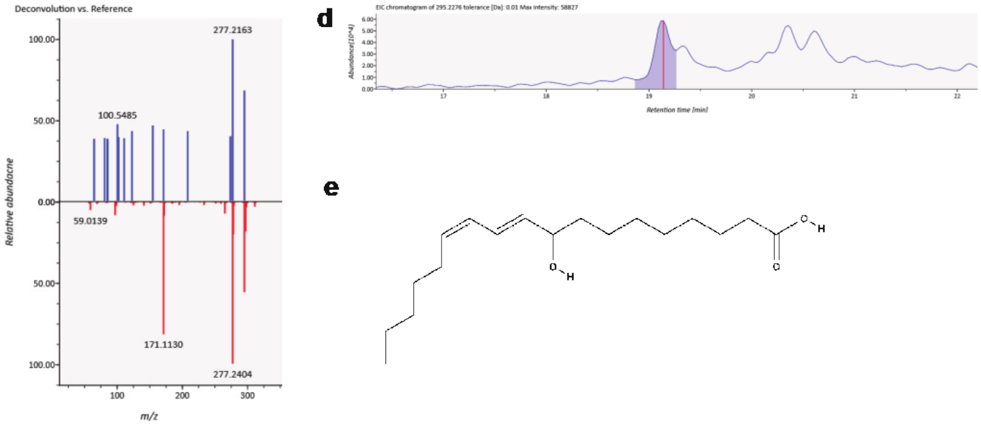

Figure S5: Fragmentation of 9-hydroxy-octadecadienoic acid. (a) LOBSTAHS and MSDIAL annotation, (b) matched fragments, (c) sample fragmentation (blue) against reference fragmentation (red), (d) EIC, and (e) compound structure.

a

LOBSTAHS Annotation	FFA 20:5 +1O (m/z = 317.2121463, RT = 18.13)
MSDIAL Annotation	11,12-epETE_mz_317.21219_RT_18.3
Ref. SMILES	CC/C=C\C/C=C\C[C@@H]1[C@@H](O1)C/C=C\C/C=C\CCCC(O)=O

b

Sample m/z	Fragment Abundance	Reference m/z	Fragment Abundance	m/z difference
59.01331	10078	59.01361465	25020770	0.000304655
61.06804	2505	61.01801682	69690.34375	0.050023185
163.14774	2817	163.1196899	9027960	0.028050059
207.13843	39081	207.1154175	1662981.875	0.02301252
208.14053	9452	208.1230011	1225704.125	0.017528901
235.97427	3773	235.9891968	22333.28711	0.014926777

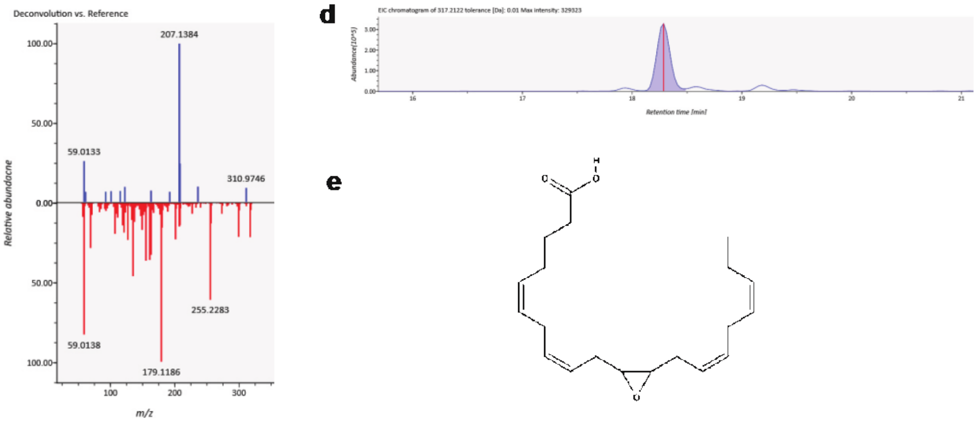

Figure S6: Fragmentation of 11,12-epoxy-eicosatetraenoic acid. (a) LOBSTAHS and MSDIAL annotation, (b) matched fragments, (c) sample fragmentation (blue) against reference fragmentation (red), (d) EIC, and (e) compound structure.

a

LOBSTAHS Annotation	FFA 20:4 +4O (m/z = 367.2119111, RT = 12.9)
**MSDIAL Annotation**	6-keto-PGE1_mz_367.21179_RT_12.901
**Ref. SMILES**	CCCCC[C@@H](/C=C/[C@H]1[C@@H](CC(=O)[C@@H]1CC(=O)CCCCC(=O)O)O)O

b

Sample m/z	Fragment Abundance	Reference m/z	Fragment Abundance	m/z difference
71.01906	2401	71.02958679	1836452.375	0.010526792
149.08028	16030	149.0882721	9750927	0.007992095
163.05661	2594	163.1020508	3058525	0.045440781

c

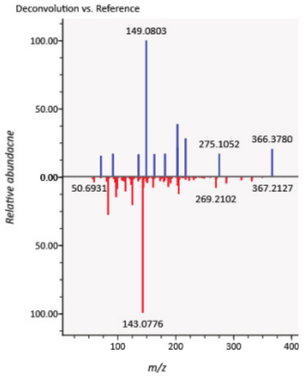

d

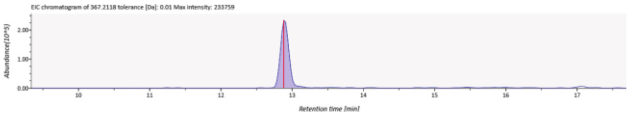

e

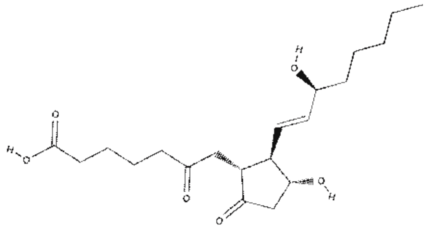

Figure S7: Fragmentation of 6-ketoprostaglandin E1. (a) LOBSTAHS and MSDIAL annotation, (b) matched fragments, (c) sample fragmentation (blue) against reference fragmentation (red), (d) EIC, and (e) compound structure.

a

LOBSTAHS Annotation	NA
**MSDIAL Annotation**	9-epHTrE (m/z = 263.16513, RT = 15.965)
**Ref. SMILES**	C=CCC=CCC1OC1CC=CCCCCC(=O)[O-]

b

Sample m/z	Fragment Abundance	Reference m/z	Fragment Abundance	m/z difference
123.08113	4105	123.0815353	5.211185932	0.000405339
137.0961	5603	137.0971832	6.915477276	0.001083228
139.11182	3127	139.1128387	1.227123737	0.001018745
165.12918	4508	165.0921021	2.226763248	0.037077949
219.17557	7985	219.1754456	20.27257156	0.000124443
263.16306	16271	263.1652832	72.97335052	0.002223203

c

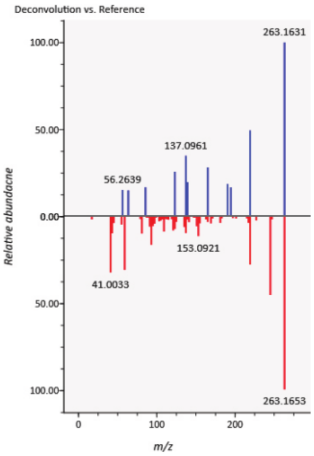

d

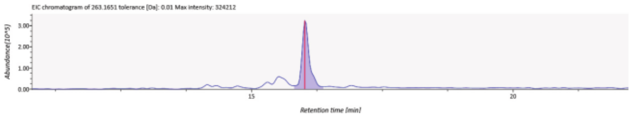

e

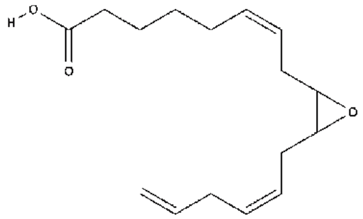

Figure S8: Fragmentation of 9-epoxy-hexadecatrienoic acid. (a) LOBSTAHS and MSDIAL annotation, (b) matched fragments, (c) sample fragmentation (blue) against reference fragmentation (red), (d) EIC, and (e) compound structure.

a

LOBSTAHS Annotation	NA
MSDIAL Annotation	6-oxoHME (m/z = 267.19632, RT = 19.494)
Ref. SMILES	CCCCCCCCC=CC(=O)CCCCC(=O)[O-]

b

Sample m/z	Fragment Abundance	Reference m/z	Fragment Abundance	m/z difference
83.04926	4855	83.05023956	6.467599392	0.000979563
85.02898	5865	85.02950287	0.161428556	0.000522869
221.18874	70095	221.1910858	3.180894375	0.002345815
249.18593	12203	249.1860046	33.57644272	7.46387E-05
267.19592	433974	267.1965637	76.22532654	0.000643721

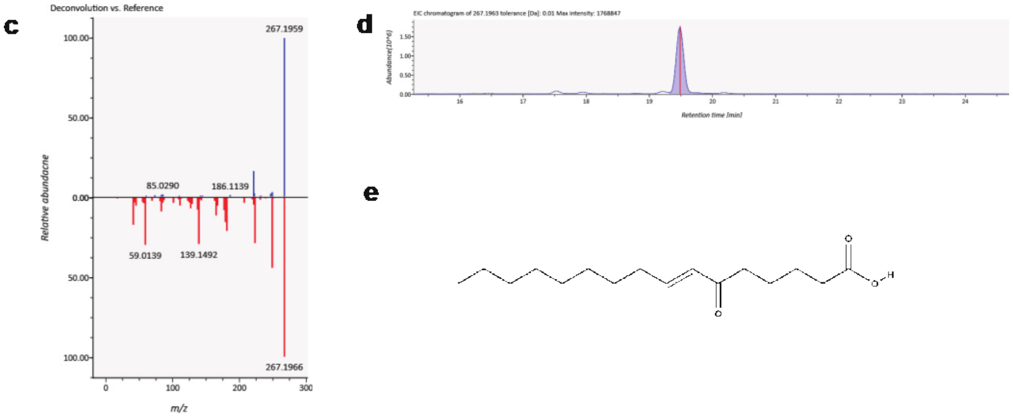

Figure S9: Fragmentation of 6-oxo-hexadecaenoic acid. (a) LOBSTAHS and MSDIAL annotation, (b) matched fragments, (c) sample fragmentation (blue) against reference fragmentation (red), (d) EIC, and (e) compound structure.

a

LOBSTAHS Annotation	NA
MSDIAL Annotation	15-deoxy-PGD2 (m/z = 333.20682, RT = 15.742)
Ref. SMILES	CCCCC/C=C/C=C/1\[C@H]([C@H](CC1=O)O)C/C=C\CCCC(=O)O

b

Sample m/z	Fragment Abundance	Reference m/z	Fragment Abundance	m/z difference
125.08761	2712	125.077095	426341.0938	0.010514968
245.1532	2770	245.15802	489861.25	0.00482002
287.22205	2930	287.2243958	169770.9063	0.002345752

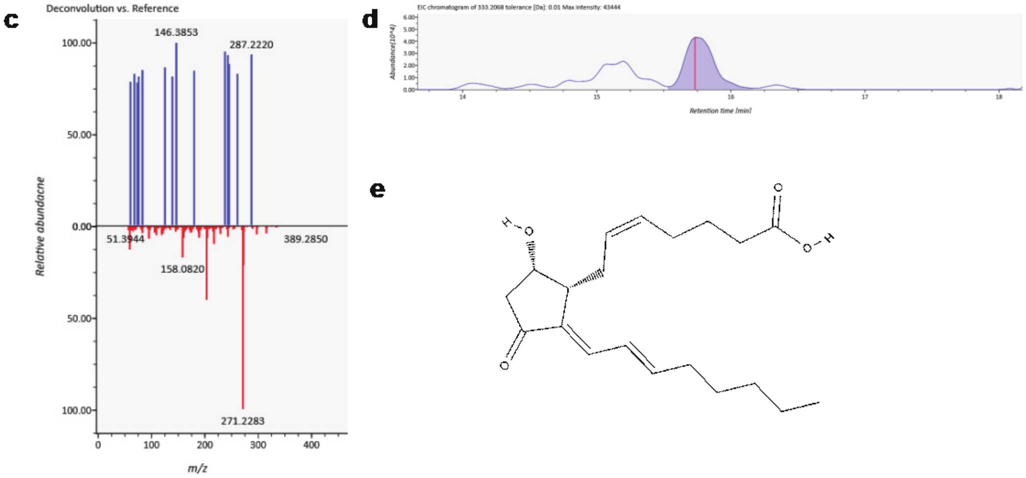

Figure S10: Fragmentation of 15-deoxyprostaglandin D2. (a) LOBSTAHS and MSDIAL annotation, (b) matched fragments, (c) sample fragmentation (blue) against reference fragmentation (red), (d) EIC, and (e) compound structure.

a	
LOBSTAHS Annotation	NA
MSDIAL Annotation	15-HpEPE (m/z = 315.19608, RT = 18.476)
Ref. SMILES	CC/C=C\C[C@H](OO)/C=C/C=C\C/C=C\C/C=C\CCCC(O)=O

b

Sample m/z	Fragment Abundance	Reference m/z	Fragment Abundance	m/z difference
59.01331	8122	59.01371384	5931934.5	0.000403837
123.08028	23768	123.0862274	478488.5625	0.005987417
125.09874	6354	125.0986797	186687.8408	6.03125E-05
147.11809	3519	147.1223145	2337663.25	0.004224453
149.0955	12250	149.1039888	1432113.25	0.008488847
151.11105	3414	151.1204378	1278157.125	0.009387822
163.11195	2557	163.1194763	3193902.75	0.007526318
179.10669	5799	179.1226807	332758.8438	0.015990664
187.11046	2599	187.1124115	206953.3125	0.001951499
189.12682	5346	189.1092987	1135993	0.017521294
191.10503	6309	191.1260988	655112.4375	0.021068833
207.13843	40566	207.1537628	103788.9219	0.015332817
217.1557	3214	217.1491241	772009.4375	0.006575854
232.99883	2488	232.9915619	349092.4063	0.00706811
271.20224	19746	271.2278611	4357482	0.025421133
272.20706	2805	272.2307434	898884.6875	0.023683408
297.18683	5290	297.2073975	1227588.625	0.020567481
315.19672	25188	315.2230225	3522142.75	0.026302481

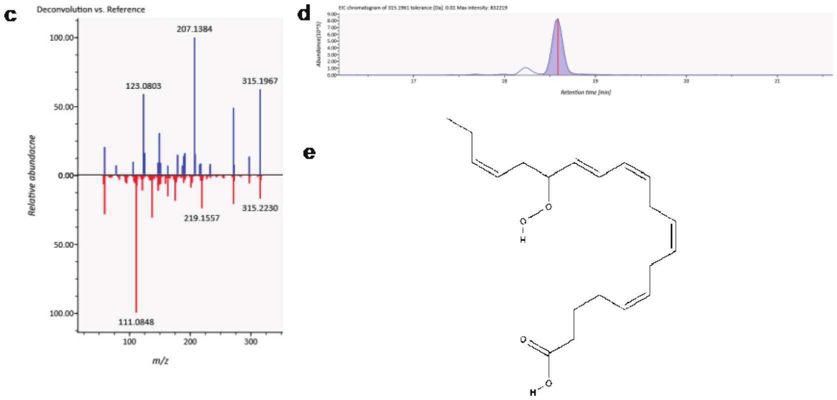

Figure S11: Fragmentation of 15-hydroperoxy-eicosapentaenoic acid. (a) LOBSTAHS and MSDIAL annotation, (b) matched fragments, (c) sample fragmentation (blue) against reference fragmentation (red), (d) EIC, and (e) compound structure.

a	
LOBSTAHS Annotation	NA
MSDIAL Annotation	5-HpEPE (m/z = 315.19598, RT = 18.957)
Ref. SMILES	CC/C=C\C/C=C\C/C=C\C/C=C\C=C\[C@@H](OO)CCCC(O)=O

b

Sample m/z	Fragment Abundance	Reference m/z	Fragment Abundance	m/z difference
59.0133	17827	59.01431856	58152032	0.001018559
71.01336	3818	71.01643372	20493372	0.003073716
106.09837	2632	106.0532074	317821.5625	0.045162603
109.06509	2837	109.0707018	9380184	0.005611599
129.05434	9049	129.0626373	31271708	0.008297329
147.11617	9030	147.1256561	21855982	0.009486128
155.06963	3298	155.0804291	13023785	0.010799077
173.13124	10707	173.1451283	20751600	0.013888343
187.15166	3223	187.1169588	280154.4375	0.034701382
201.16472	9038	201.179184	18088456	0.01446398

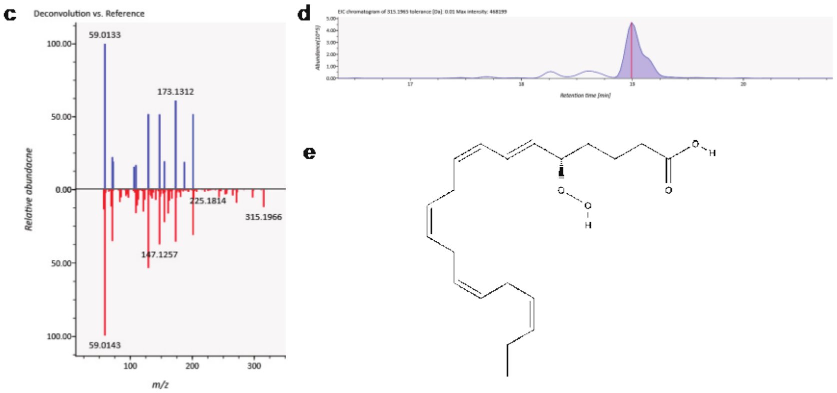

Figure S12: Fragmentation of 5-hydroperoxy-eicosapentaenoic acid. (a) LOBSTAHS and MSDIAL annotation, (b) matched fragments, (c) sample fragmentation (blue) against reference fragmentation (red), (d) EIC, and (e) compound structure.

a	
LOBSTAHS Annotation	NA
MSDIAL Annotation	5-HEPE (m/z = 317.21194, RT = 18.781)
Ref. SMILES	CC/C=C\C/C=C\C/C=C\C/C=C\C=C/C(O)CCCC(=O)O

b

Sample m/z	Fragment Abundance	Reference m/z	Fragment Abundance	m/z difference
59.0133	10044	59.01368332	46642464	0.000383319
67.88551	2643	67.84736633	8798.493164	0.038143667
111.07948	2802	111.0583191	1067545.125	0.021160908
115.03925	14749	115.0438919	92089336	0.004641907
191.98151	2547	191.9942474	22595.75781	0.012737437
255.20895	3014	255.232254	13771952	0.023304028

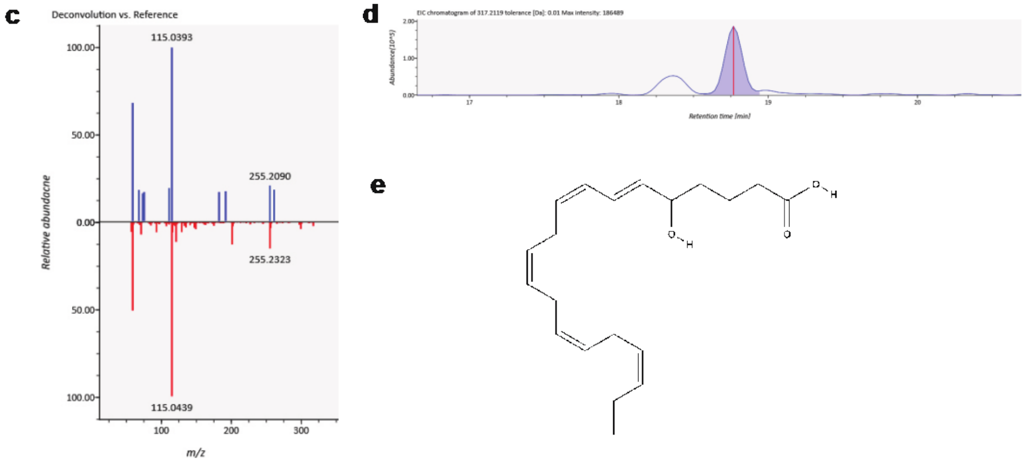

Figure S13: Fragmentation of 5-hydroxy-eicosapentaenoic acid. (a) LOBSTAHS and MSDIAL annotation, (b) matched fragments, (c) sample fragmentation (blue) against reference fragmentation (red), (d) EIC, and (e) compound structure.

a	
LOBSTAHS Annotation	FFA 16:3 (m/z = 249.1861468, RT = 20.88)
MSDIAL Annotation	Hexadecatrienoic acid; (m/z = 249.18582, RT = 20.87)
Ref. SMILES	CCCC=CCC=CCC=CCCCCC(=O)O

b

Sample m/z	Fragment Abundance	Reference m/z	Fragment Abundance	m/z difference
59.01346	4213	59.01385117	17.85000038	0.000391166
231.17738	3439	231.1754456	4.980000019	0.001934443
249.1487	4970	249.1860046	100	0.037304639

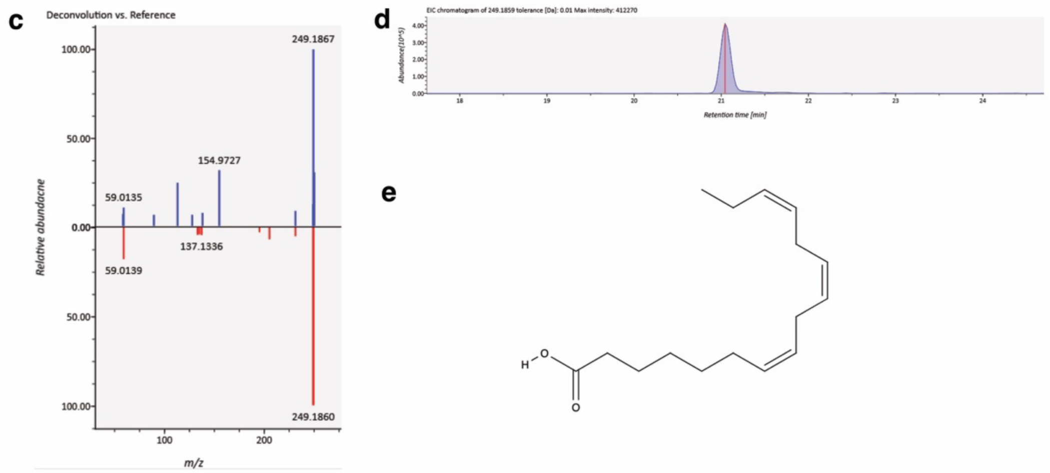

Figure S14: Fragmentation of Hexadecatrienoic acid. (a) LOBSTAHS and MSDIAL annotation, (b) matched fragments, (c) sample fragmentation (blue) against reference fragmentation (red), (d) EIC, and (e) compound structure.

a

LOBSTAHS Annotation	FFA 18:0 (m/z = 283.2639462, RT = 26.57)
MSDIAL Annotation	Stearic acid; (m/z = 283.26395, RT = 26.602)
Ref. SMILES	CCCCCCCCCCCCCCCCCC(=O)O

b

Sample m/z	Fragment Abundance	Reference m/z	Fragment Abundance	m/z difference
59.0133	6989	59.01385117	7.155142307	0.000551166
265.2514	7203	265.2536926	26.12514114	0.002292627
283.26462	2674973	283.2642517	44.88751221	0.000368291

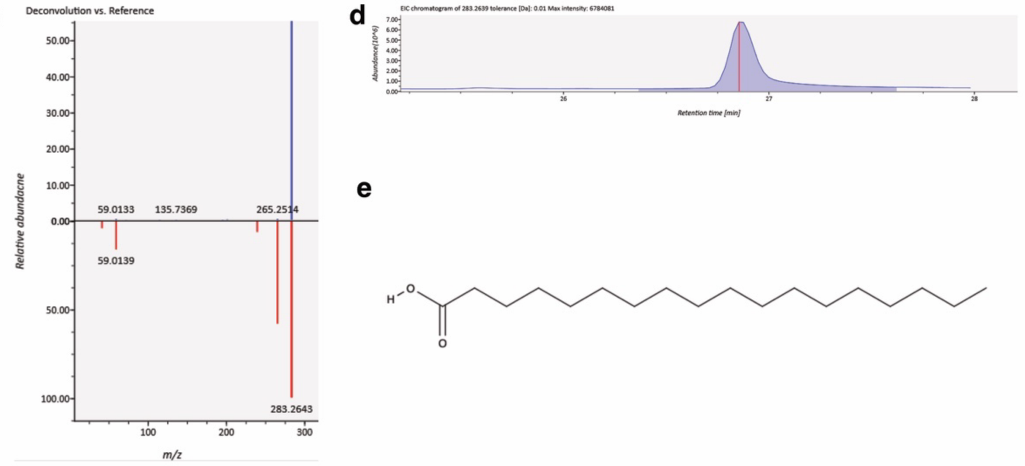

Figure S15: Fragmentation of Stearic acid. (a) LOBSTAHS and MSDIAL annotation, (b) matched fragments, (c) sample fragmentation (blue) against reference fragmentation (red), (d) EIC, and (e) compound structure.

a

LOBSTAHS Annotation	FFA 20:5 (m/z = 301.2172014, RT = 22.54)
MSDIAL Annotation	EPA; (m/z = 301.21716, RT = 22.563)
Ref. SMILES	CC/C=C/C/C=C/C/C=C/C/C=C/C/C=C/CCCC(=O)O

b

Sample m/z	Fragment Abundance	Reference m/z	Fragment Abundance	m/z difference
59.01315	9711	59.01366806	26290800	0.00051806
203.17883	3427	203.1911469	4464962	0.012316851
257.22464	8333	257.2481079	10982313	0.02346791
301.21414	18496	301.2416077	17291350	0.027467666

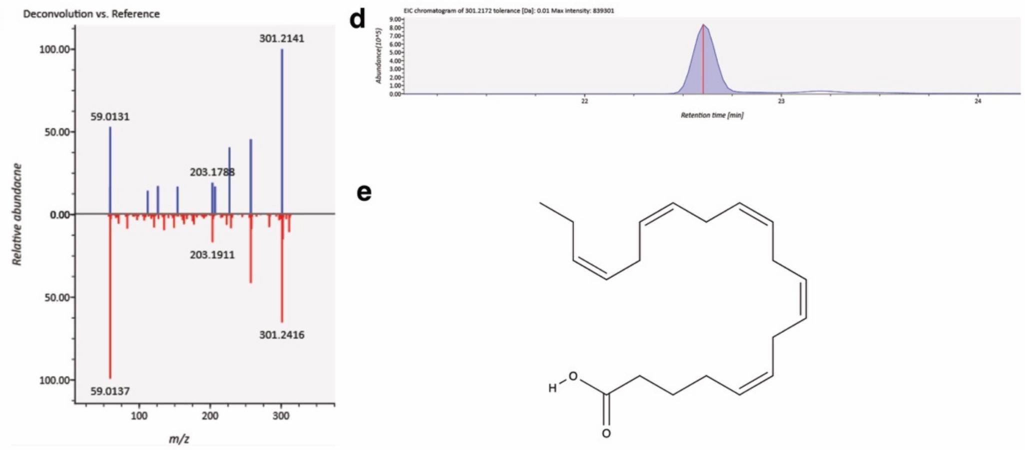

Figure S16: Fragmentation of Eicosapentaenoic acid. (a) LOBSTAHS and MSDIAL annotation, (b) matched fragments, (c) sample fragmentation (blue) against reference fragmentation (red), (d) EIC, and (e) compound structure.

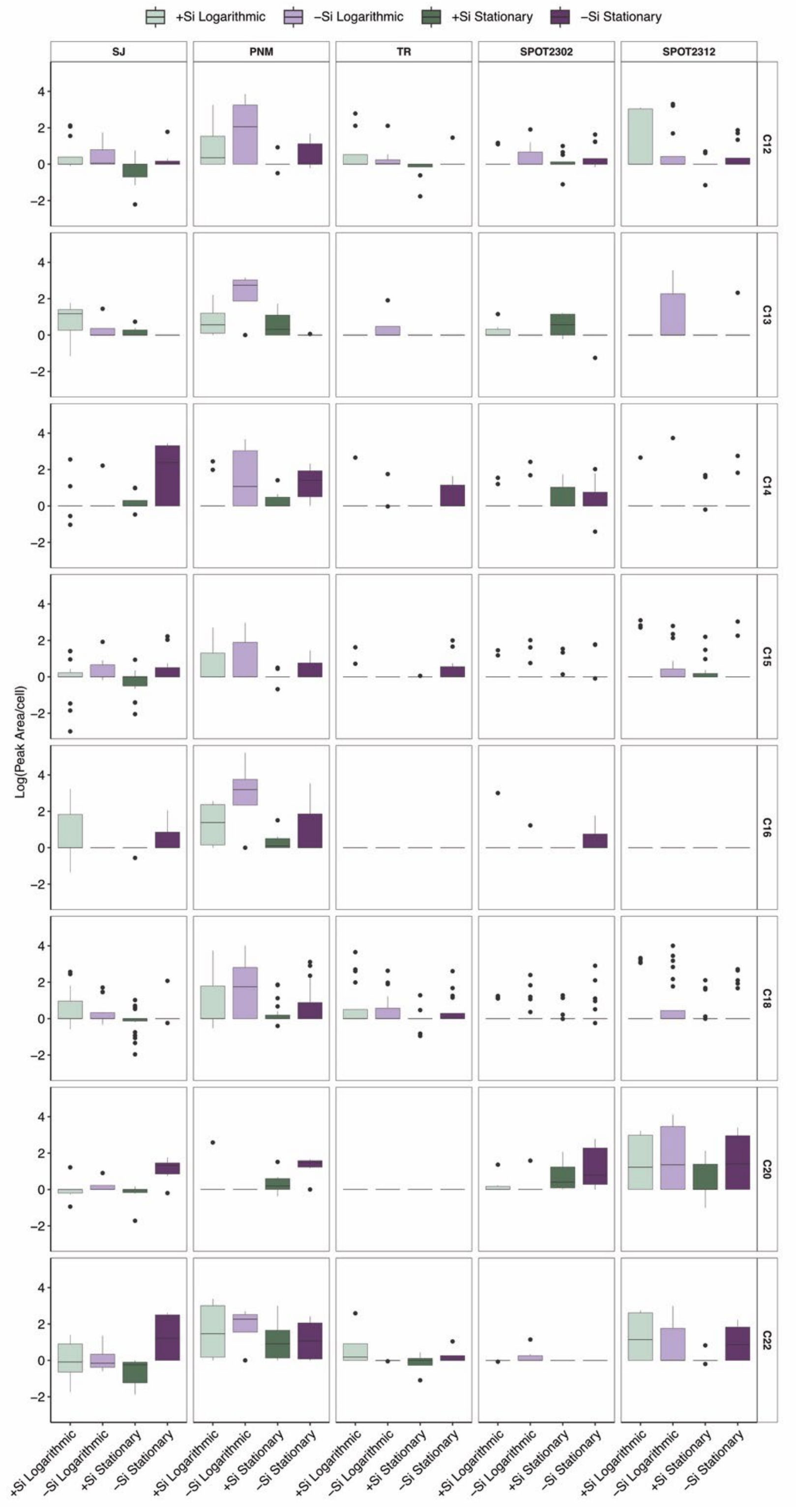

Figure S17: Boxplots of oxylipin peak areas (cell normalized and log transformed) separated by diatom species (*S. japonicum*, *P. multiseries*, *T. rotula*, SPOT2302, SPOT2312) and by carbon chain length. Boxplots are colored by culture condition: +Si Log (light green), -Si Log (light purple), +Si Stat (green), -Si Stat (purple).

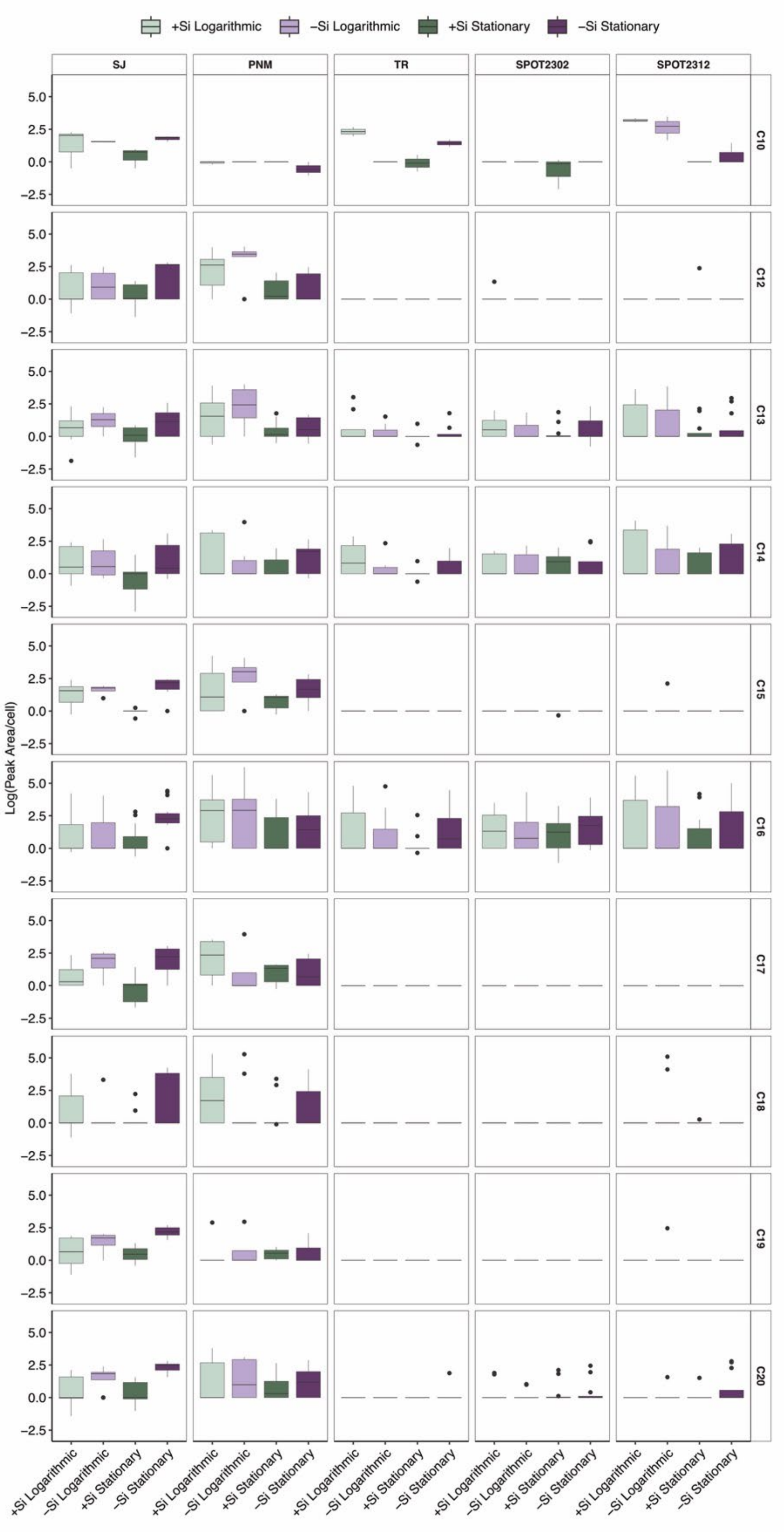

Figure S18: Boxplots of free fatty acid peak areas (cell normalized and log transformed) separated by diatom species (*S. japonicum*, *P. multiseries*, *T. rotula*, SPOT2302, SPOT2312) and by carbon chain length. Boxplots are colored by culture condition: +Si Log (light green), -Si Log (light purple), +Si Stat (green), -Si Stat (purple).

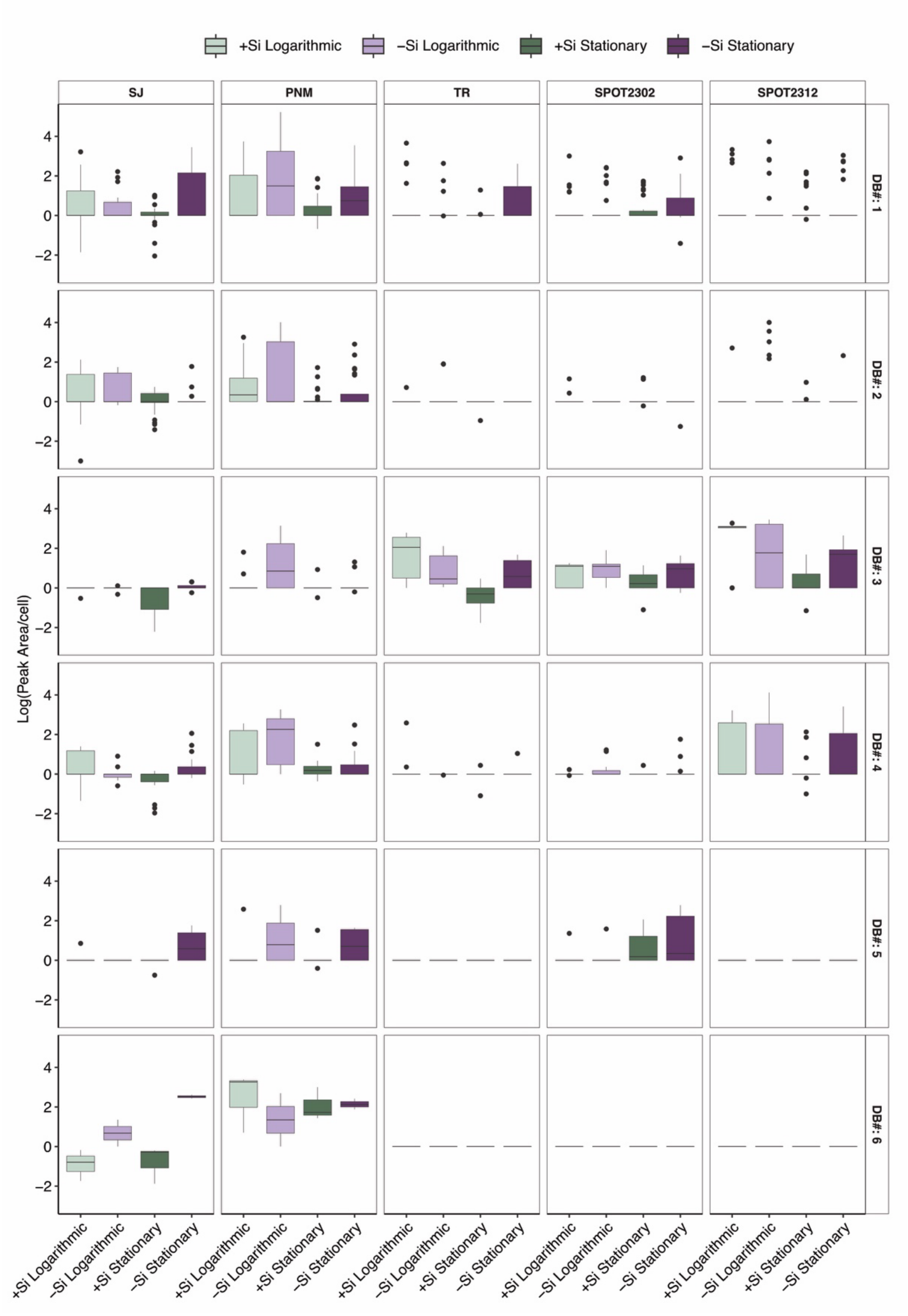

Figure S19: Boxplots of oxylipin peak areas (cell normalized and log transformed) separated by diatom species (*S. japonicum*, *P. multiseries*, *T. rotula*, SPOT2302, SPOT2312) and by degree of saturation. Boxplots are colored by culture condition: +Si Log (light green), -Si Log (light purple), +Si Stat (green), -Si Stat (purple).

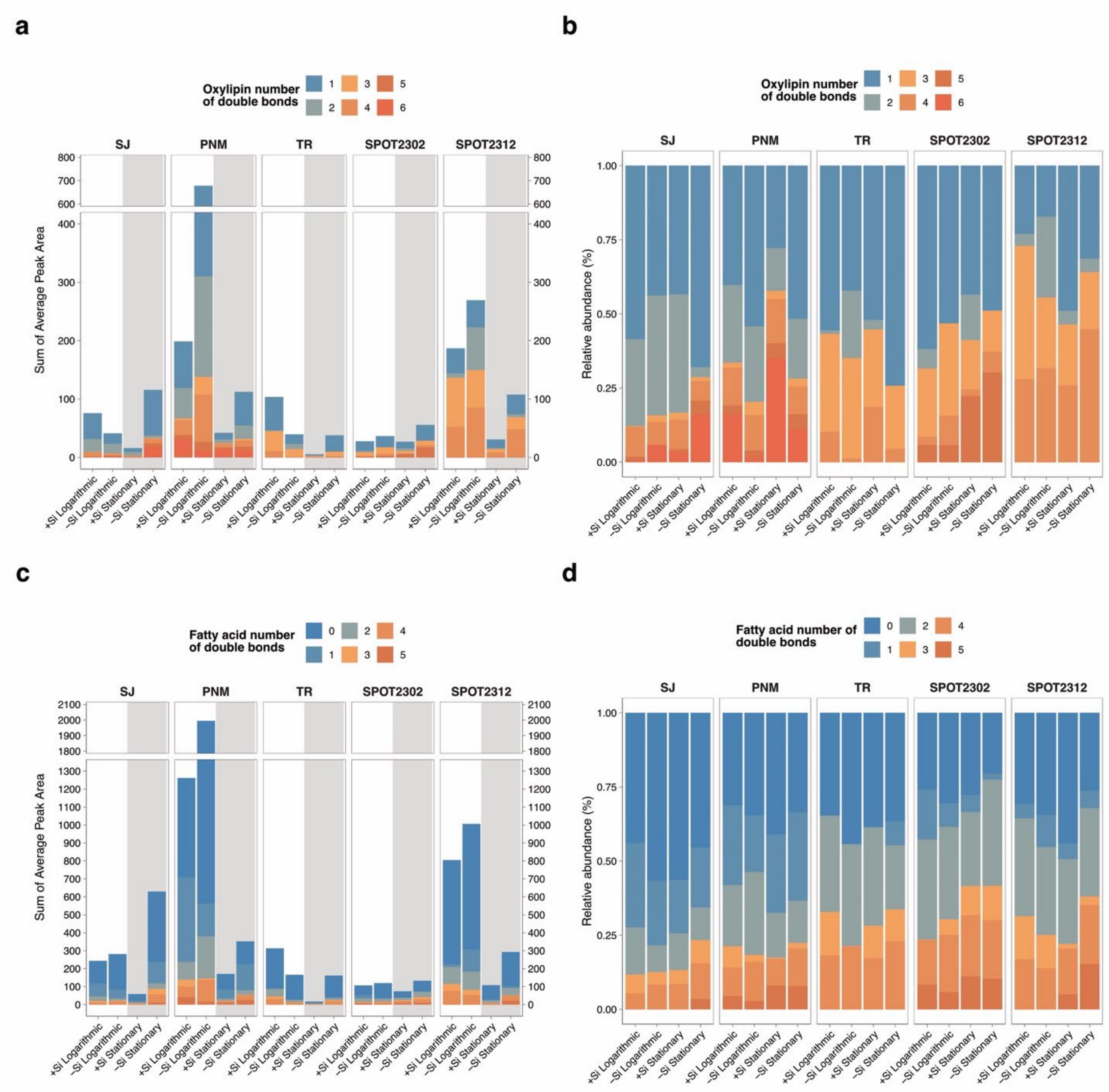

Figure S20: Degree of saturation for oxylipins and fatty acids: (a) Sum of cell normalized and square root transformed peak areas for oxylipins, (b) Relative abundance for oxylipins, (c) Sum of cell normalized and square root transformed peak areas for fatty acids, (d) Relative abundance for fatty acids.

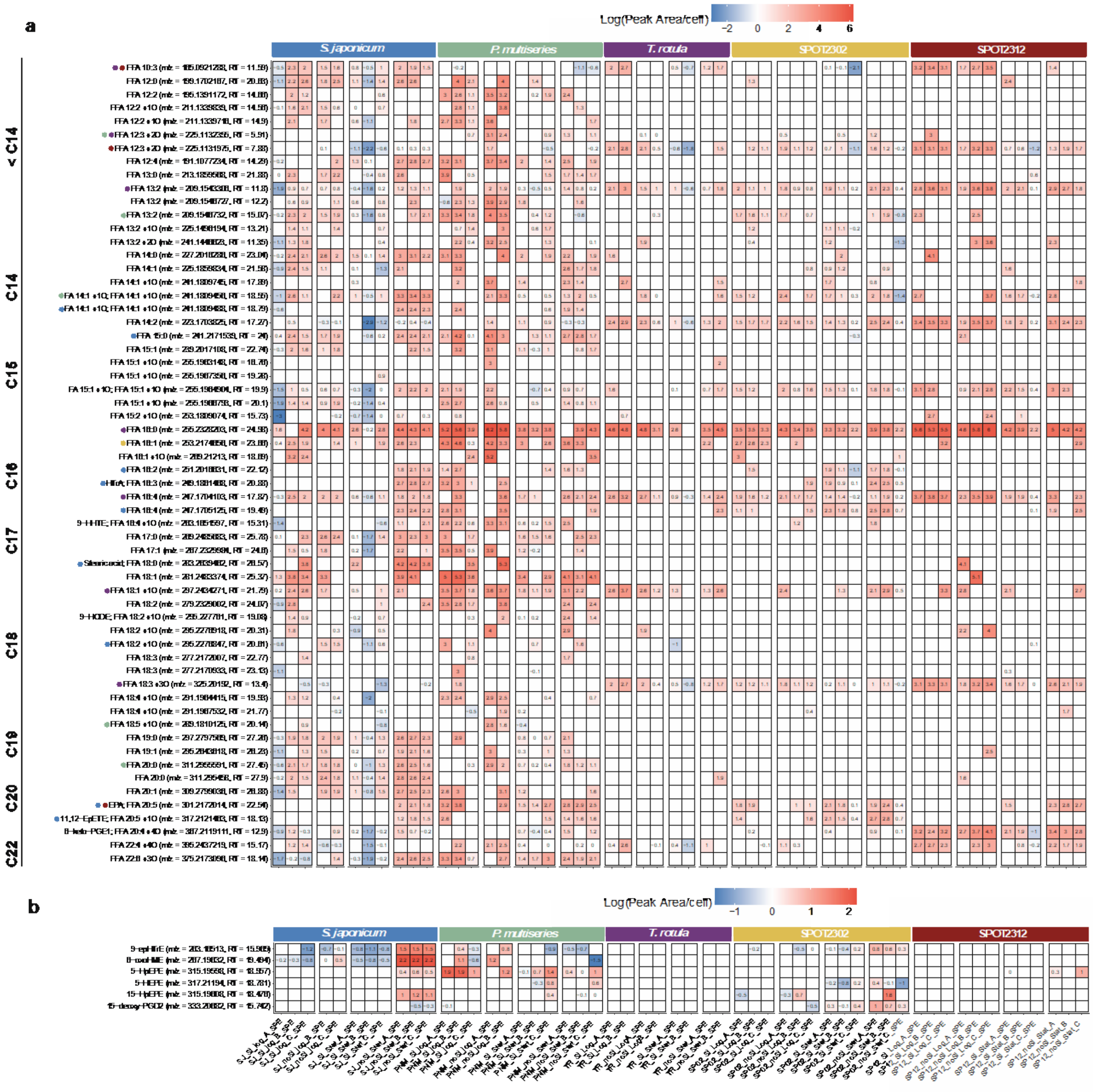

Figure S21: Cell normalized and log transformed peak area for each feature: (a) LOBSTAHS annotated; (b) MSDIAL annotated. Cell normalized and log transformed values are displayed in black text for their respective sample and feature. Samples that lacked a feature are denoted by empty white boxes. Features are organized based on carbon chain length, retention time, degree of saturation, and degree of oxidation; samples are organized based on species and culture condition. Significant features from ANOVA are denoted by circles with the respective species colors: (blue) S. japonicum, (green) P. multiseries, (purple) T. rotula, (yellow) SPOT2302, (orange) SPOT2312.

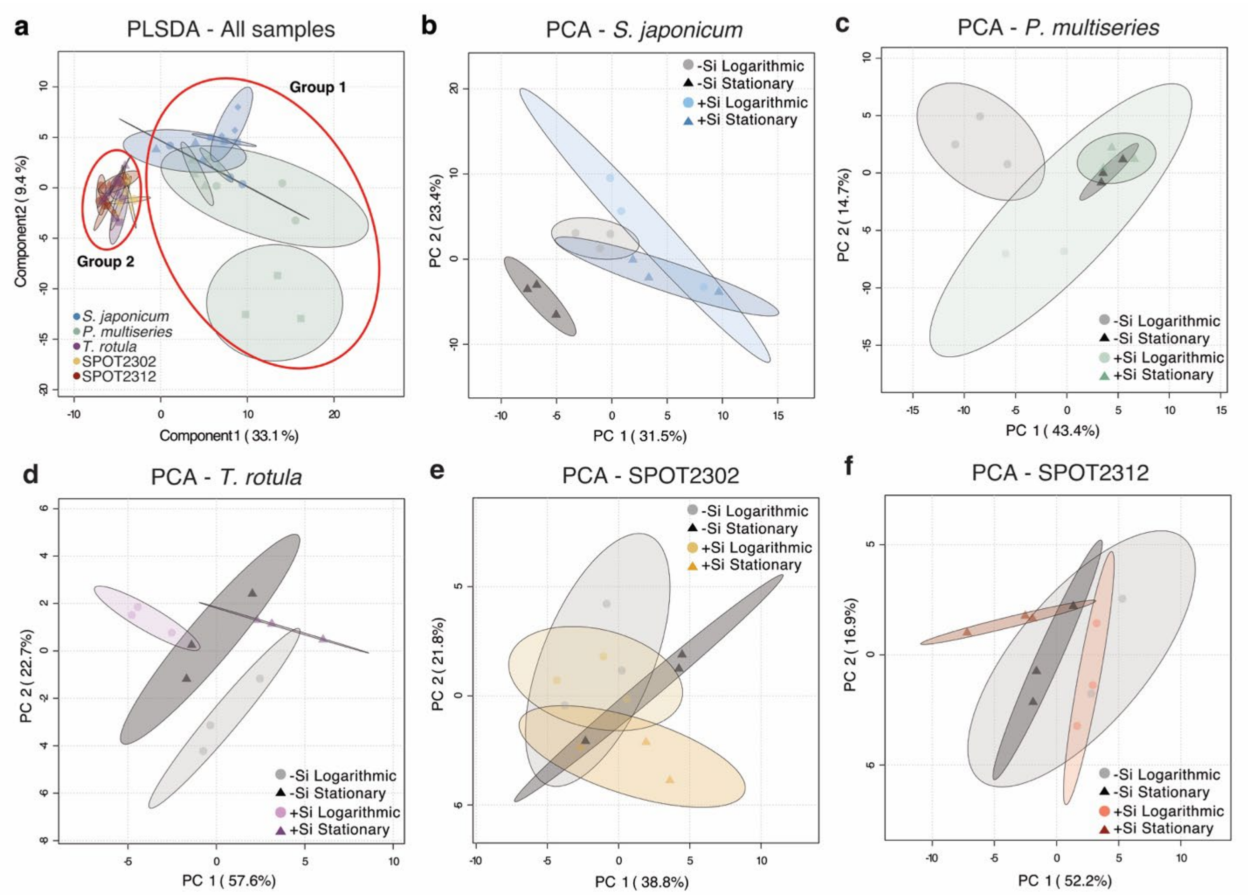

Figure S22: Score plots generated from partial least squares discriminant analysis (PLS-DA) and principal component analysis (PCA) on all features annotated by LOBSTAHS (n=60) and their cell normalized and log transformed peak areas: (a) PLS-DA on all samples; (b) PCA on samples from Skeletonema japonicum (Si-replete, blue); (c) PCA on samples from Pseudo-nitzschia multiseries (Si-replete, green); (d) PCA on samples from Thalassiosira rotula (Si-replete, purple); (e) PCA on samples from the SPOT2302 isolate (Si-replete, yellow); (f) PCA on samples from the SPOT2312 isolate (Si-replete, red). Si-limited samples collected during logarithmic growth are colored gray, whereas those collected during stationary growth are colored black.

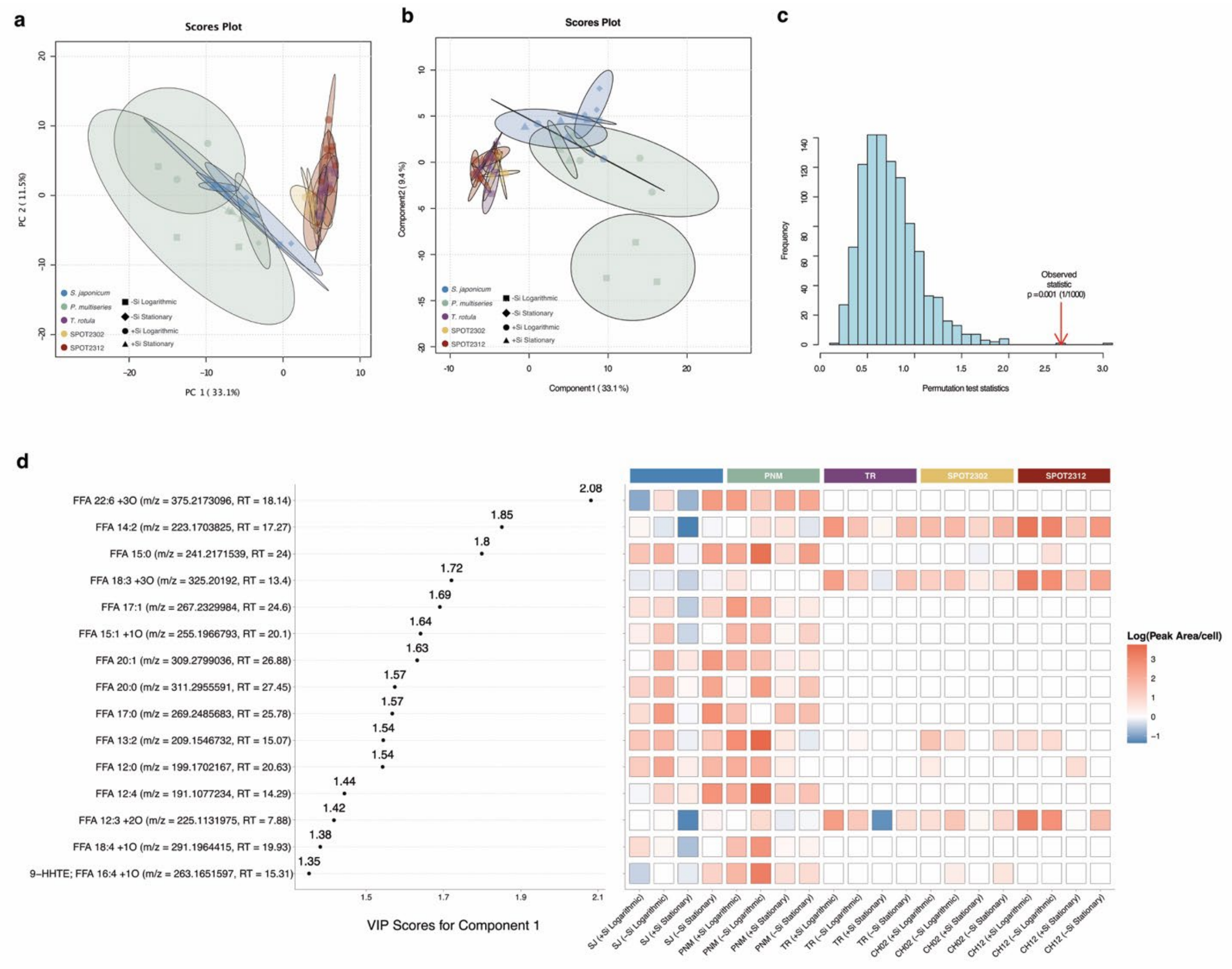

Figure S23: Comparison between PCA and PLSDA using all features (n = 60). (a) PCA on all samples, (b) PLSDA on all samples, (c) Permutation for PLSDA, (d) VIP Scores from PLSDA alongside their respective cell normalized and log transformed peak areas.

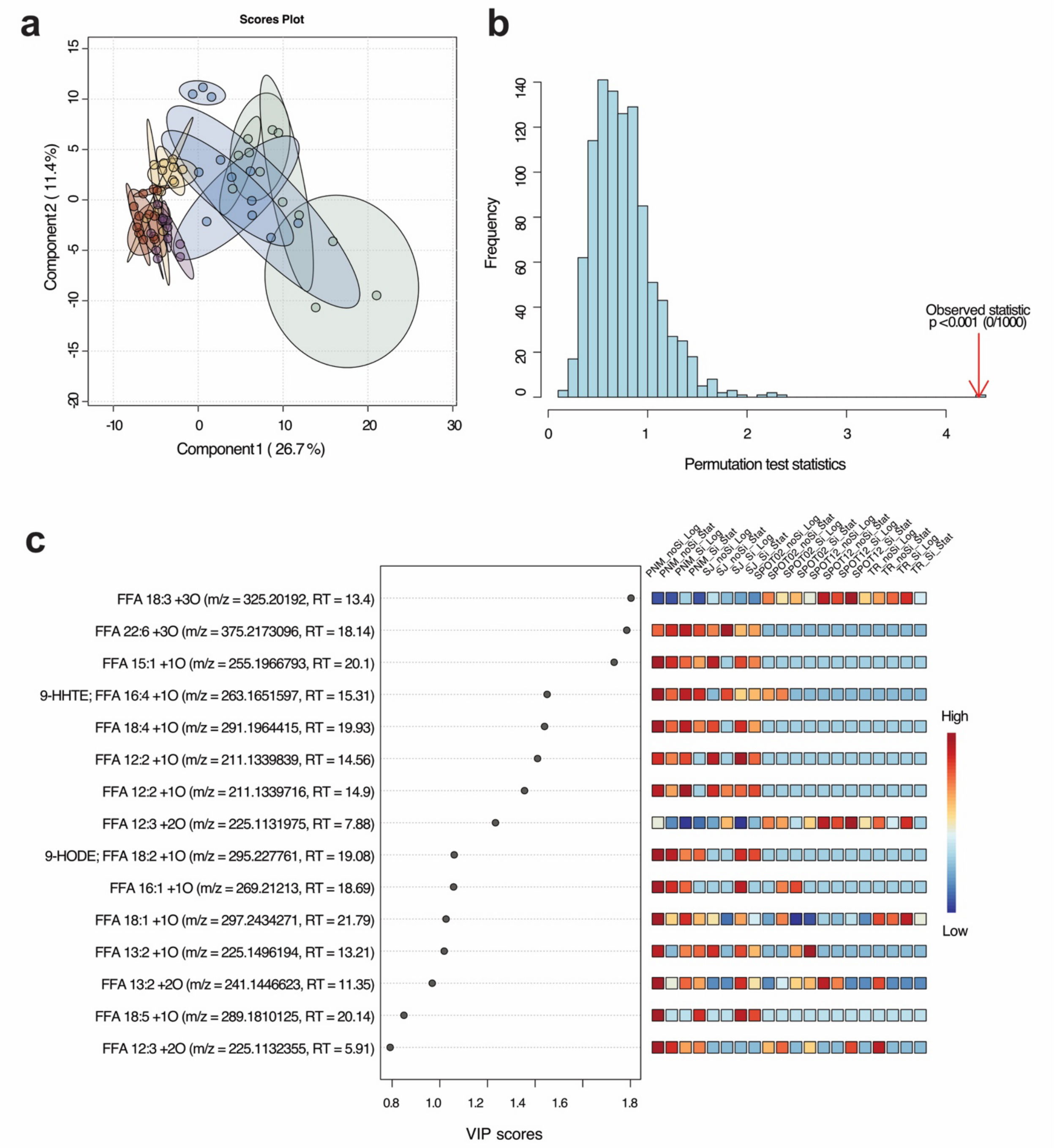

Figure S24: PLSDA on features annotated as LOFAs (n = 28). (a) PLS-DA, (b) Permutation test, (c) VIP Scores from PLSDA alongside their respective log-transformed, cell normalized and peak areas.

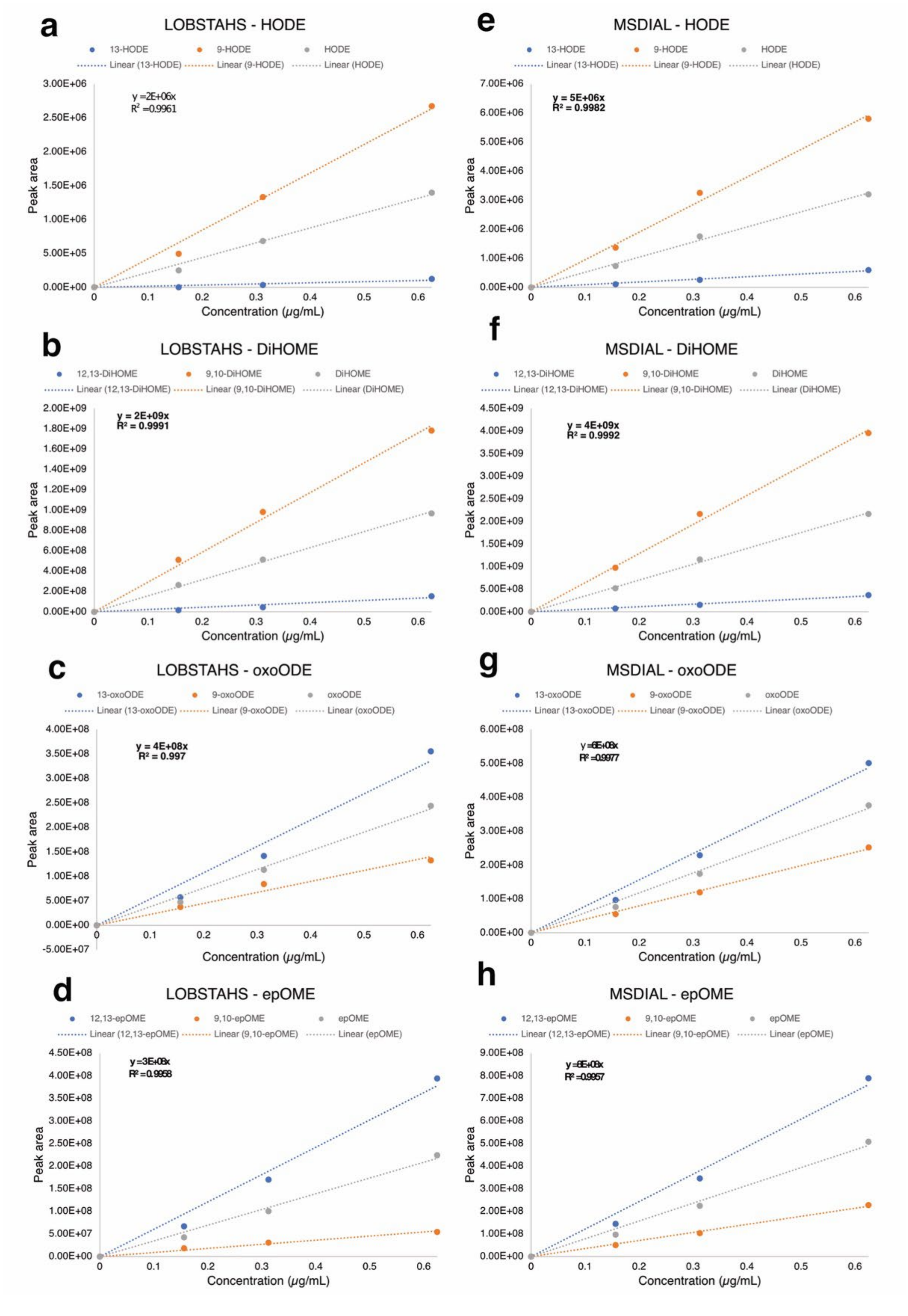

Figure S25: Standard Curves based of C18 LOFAs.
